# Subsequent AS01-adjuvanted vaccinations induce similar transcriptional responses in populations with different disease statuses

**DOI:** 10.1371/journal.pone.0276505

**Published:** 2022-11-10

**Authors:** Margherita Coccia, Wivine Burny, Marie-Ange Demoitié, Paul Gillard, Robert A. van den Berg, Robbert van der Most

**Affiliations:** 1 GSK, Rixensart, Belgium; 2 GSK, Wavre, Belgium; 3 GSK, Rockville, MD, United States of America; IAVI, UNITED STATES

## Abstract

Transcriptional responses to adjuvanted vaccines can vary substantially among populations. Interindividual diversity in levels of pathogen exposure, and thus of cell-mediated immunological memory at baseline, may be an important determinant of population differences in vaccine responses. Adjuvant System AS01 is used in licensed or candidate vaccines for several diseases and populations, yet the impact of pre-existing immunity on its adjuvanticity remains to be elucidated. In this exploratory post-hoc analysis of clinical trial samples (clinicalTrials.gov: NCT01424501), we compared gene expression patterns elicited by two immunizations with the candidate tuberculosis (TB) vaccine M72/AS01, between three groups of individuals with different levels of memory responses to TB antigens before vaccination. Analyzed were one group of TB-disease-treated individuals, and two groups of TB-disease-naïve individuals who were (based on purified protein derivative [PPD] skin-test results) stratified into PPD-positive and PPD-negative groups. Although TB-disease-treated individuals displayed slightly stronger transcriptional responses after each vaccine dose, functional gene signatures were overall not distinctly different between groups. Considering the similarities with the signatures found previously for other AS01-adjuvanted vaccines, many features of the response appeared to be adjuvant-driven. Across groups, cell proliferation-related signals at 7 days post-dose 1 were associated with increased anti-M72 antibody response magnitudes. These early signals were stronger in the TB-disease-treated group as compared to both TB-disease-naïve groups. Interindividual homogeneity in gene expression levels was also higher for TB-disease-treated individuals post-dose 1, but increased in all groups post-dose 2 to attain similar levels between the three groups. Altogether, strong cell-mediated memory responses at baseline accelerated and amplified transcriptional responses to a single dose of this AS01-adjuvanted vaccine, resulting in more homogenous gene expression levels among the highly-primed individuals as compared to the disease-naïve individuals. However, after a second vaccination, response heterogeneity decreased and was similar across groups, irrespective of the degree of immune memory acquired at baseline. This information can support the design and analysis of future clinical trials evaluating AS01-adjuvanted vaccines.

## Introduction

The biologic understanding of a vaccine’s immunogenicity, or lack thereof, within a given population is frequently limited by the numerous host characteristics that represent a correlate of vaccine responsiveness. These factors can include, amongst others, race, sex, polymorphisms of the human leukocyte antigen or toll-like receptors (TLRs), and/or differences in the level of pathogen priming incurred by the host [1–3]. Collectively these factors can lead to adaptive vaccine responses that vary significantly across individuals, even if the vaccinees are healthy. Since vaccine adjuvants are used to selectively stimulate different routes of innate signaling, which then translate into enhanced adaptive immunity, the choice of adjuvant can affect a vaccine’s ability to consistently elicit robust immune responses across populations with different levels of responsiveness [4].

Adjuvant System AS01 contains the TLR-4 ligand MPL (3-*O*-desacyl-4′-monophosphoryl lipid A) and QS-21, a saponin extracted from the bark of the *Quillaja saponaria* Molina tree, and liposomes [5, 6]. Multiple studies evaluating licensed or candidate AS01-adjuvanted vaccines demonstrated the potential of these vaccines to provide critical public health benefits in populations with different exposure histories [7, 8]. These studies demonstrated a high (≥ 90%) efficacy in older adults who received the licensed AS01-adjuvanted recombinant herpes zoster vaccine, and partial efficacy in naïve infants and primed older children who received the licensed malaria vaccine RTS,S/AS01 [9–11]. Partial efficacy was also recently reported for the M72/AS01 candidate tuberculosis (TB) vaccine, when administered to adults in TB-endemic regions who had been infected with TB’s etiologic agent *Mycobacterium tuberculosis* (Mtb), as determined by interferon [IFN]-γ release assay [12, 13]. Furthermore, AS01-adjuvanted hepatitis B surface antigen (HBs) vaccines (HBs/AS01) were found able to elicit robust innate and adaptive immune responses in HBs-naïve adults [14–18]. Collectively, these studies conducted in populations with variable degrees of baseline immune priming indicated, first, that AS01-adjuvanted vaccines were immunogenic in these populations independent of the immune background, and second, that qualitative and quantitative differences in the immune responses may exist between populations with diverse levels of immune priming, including naïve individuals. There is thus a need to increase our understanding of the effect of pre-existing immunological memory on the variability in the immune responses to these vaccines, to inform future immunization strategies for AS01-adjuvanted vaccines in post-exposure settings.

Systems vaccinology represents a valuable tool for such assessments, as it can provide deep insight into mechanisms controling vaccine immunogenicity, by quantifying the levels of engagement of specific pathways or gene signatures that are associated with adaptive immunity. Intriguingly, we previously detected several similarities in whole-blood (WB) or peripheral blood mononuclear cell (PBMC)-derived gene signatures elicited by either HBs/AS01 or RTS,S/AS01 in HBs- or malaria-naïve adults, respectively, or by M72/AS01 in Bacille Calmette-Guérin (BCG)-positive adults [17, 19, 20]. These signatures were characterized by positive regulation of IFN- and innate-cell-related genes, and negative regulation of natural killer (NK)-associated genes, and were detectable one day after the first or second vaccination. We therefore hypothesized that the gene signatures elicited by any AS01-adjuvanted vaccine were at least partially determined by the transcriptional responses to the immune stimulants contained in this Adjuvant System, and that transcriptional responses found for one AS01-adjuvanted vaccine might thus to some extent be generalizable to other similarly formulated vaccines.

We evaluated the effects of pre-existing immunological memory on the magnitudes, functionalities, and inter-subject variability of the transcriptional responses to a clinically relevant AS01-adjuvanted vaccine. As a working model, we focused on responses to M72/AS01 observed in adults who had acquired diverse degrees of Mtb priming at baseline. A driver for this selection was the unique availability of PBMC samples previously collected in the context of a Phase II trial, which evaluated the safety and adaptive immunogenicity of M72/AS01 in adult populations with different histories of TB-disease exposure [21]. TB represents a relevant disease model for such studies due to its complex host response spectrum, which translates into diverse clinical statuses within infected populations. This spectrum spans from clinically asymptomatic and non-transmissible latent TB infection (LTBI) to active TB disease that is transmissible in active pulmonary TB [22]. The bulk of the transmission is mediated by adolescents and adults. Given that one quarter of the global population is Mtb-infected, with approximately 6 million new cases and 1.5 million deaths in 2020 [23], TB constitutes a vast medical need. Though several transcriptomic biomarkers of TB disease stages and/or risks have been found [24–28], any protective gene signatures or other correlates of protection have yet to be identified [29]. Still, recent data of the Phase IIb M72/AS01 trial demonstrated 54% efficacy in preventing active pulmonary TB in adults with LTBI [13], which is the first evidence of vaccine-mediated protection against the progression from LTBI to active disease. This is hopeful considering that the expected public health impact of a disease-preventing TB vaccine targeted at adults and adolescents is considered superior (assuming equal vaccine efficacy) to that of a new vaccine replacing neonatal BCG administration [30]. Currently, BCG is still the only licensed TB vaccine.

**Fig 1:**
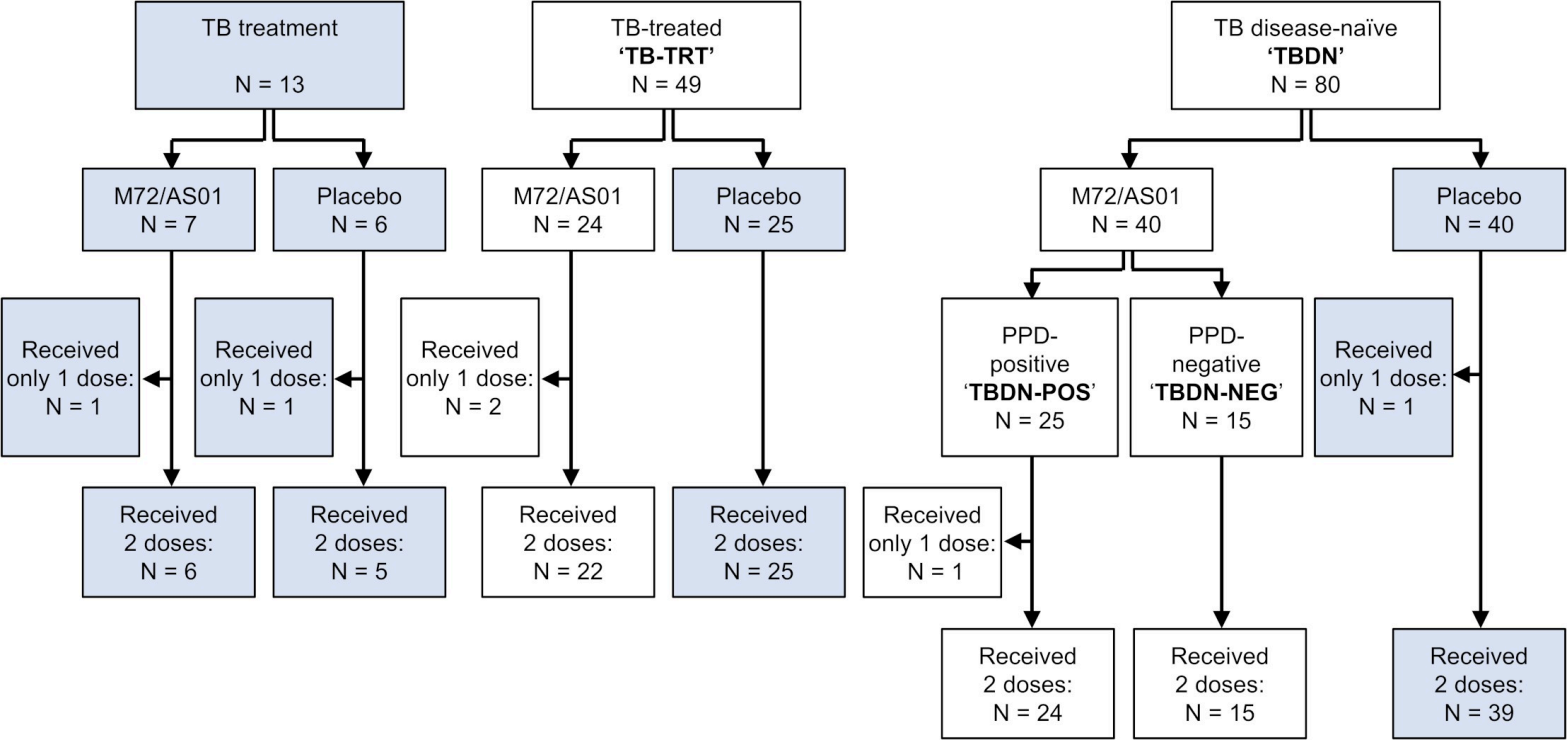
Participant flow diagram. Numbers of subjects from three previously described cohorts [21] with samples that were either included (white boxes) or excluded (grey boxes) in the current analyses, are shown. The tuberculosis (TB) treatment cohort included adults who were receiving treatment for TB disease and had completed the intensive phase of the treatment. The TB-treated (TB-TRT) cohort included adults with a previous history of successfully treated pulmonary TB disease at least 1 year before vaccination (see ref. [21] and Materials and Methods for cohort details). The TB disease-naïve (TBDN) cohort included adults with no history of TB disease. Subjects were excluded from the current analyses either due to a small sample size (TB treatment cohort), or because the intervention was out of scope for the current objective (all three placebo groups). The current analyses were performed on the group of TB-TRT vaccinees, and on two groups of TBDN vaccinees who were considered either purified protein derivative (PPD)-positive or PPD-negative based on an induration size of ≥ 10 mm or < 10 mm, respectively, in the tuberculin skin test performed at baseline (the TBDN-POS and TBDN-NEG groups, respectively). N, number of participants.

The present exploratory post-hoc analysis utilized samples from M72/AS01 vaccinees only, derived from two of the three initial study cohorts of the Phase II trial [21]. A third, incompletely recruited cohort of subjects receiving treatment for active TB was not analyzed due to its small sample size (**Fig 1**). Analyzed subsets comprised M72/AS01 vaccinees who were either successfully treated for pulmonary TB disease, or TB-disease-naïve (i.e., expected to have had no encounters with symptomatic TB disease as inferred by a negative chest X-ray [21]). The TB-disease-naïve vaccinees were further stratified into two groups based on their purified protein derivative (PPD) tuberculin skin test (TST) results [21], lacking widely available, more sensitive and specific diagnostic methods to detect LTBI [31, 32]. The transcriptional responses to M72/AS01 were then compared across the resulting three groups of subjects. The TST detects Mtb sensitization via a delayed-type hypersensitivity response provoked by cell-mediated immunity (CMI) to Mtb antigens, including the Mtb39A and Mtb32A components of the M72 recombinant antigen. Thus, this design allowed us to investigate the effect of CMI-mediated immunological memory on AS01-induced gene expression patterns. Evaluations versus baseline were performed 7 or 30 days after the first vaccine dose, and 7 days after the second dose. The rationale for selecting these timepoints rather than the transcriptional response peaks—which for this and other AS01-adjuvanted vaccines typically occur 6–24 h after vaccination [14, 15, 17]—was based on observations made for BCG-primed, Mtb-infection-naïve and TB-disease-naïve M72/AS01 vaccinees [19]. These data showed that a combination of a timepoint before vaccination with a timepoint 7 days after each vaccination best captures the vaccine-induced gene expression, including both the tail-ends of the signals driven by the adjuvant AS01, and the signals reflecting the initiation of the adaptive immune response. This allowed the identification of potential clinically relevant transcriptional responses to the vaccine. For instance, analysis at D37 in that study enabled the detection of a key gene signature driven by AS01 [19]. Moreover, inter-subject variability in gene expression elicited by this vaccine was greater at ~1 week vs 1 day postvaccination [10], suggesting that the granularity of data informing on potentially protective signatures may be higher when derived from such later timepoints as compared to innate immunity peak timepoints.

The presented results will inform downstream research and development of AS01-adjuvanted vaccines, including selection of immunization regimens, to benefit future vaccine studies in populations with different levels of baseline exposure.

## Results

### Group characterization and adaptive response

**Fig 2:**
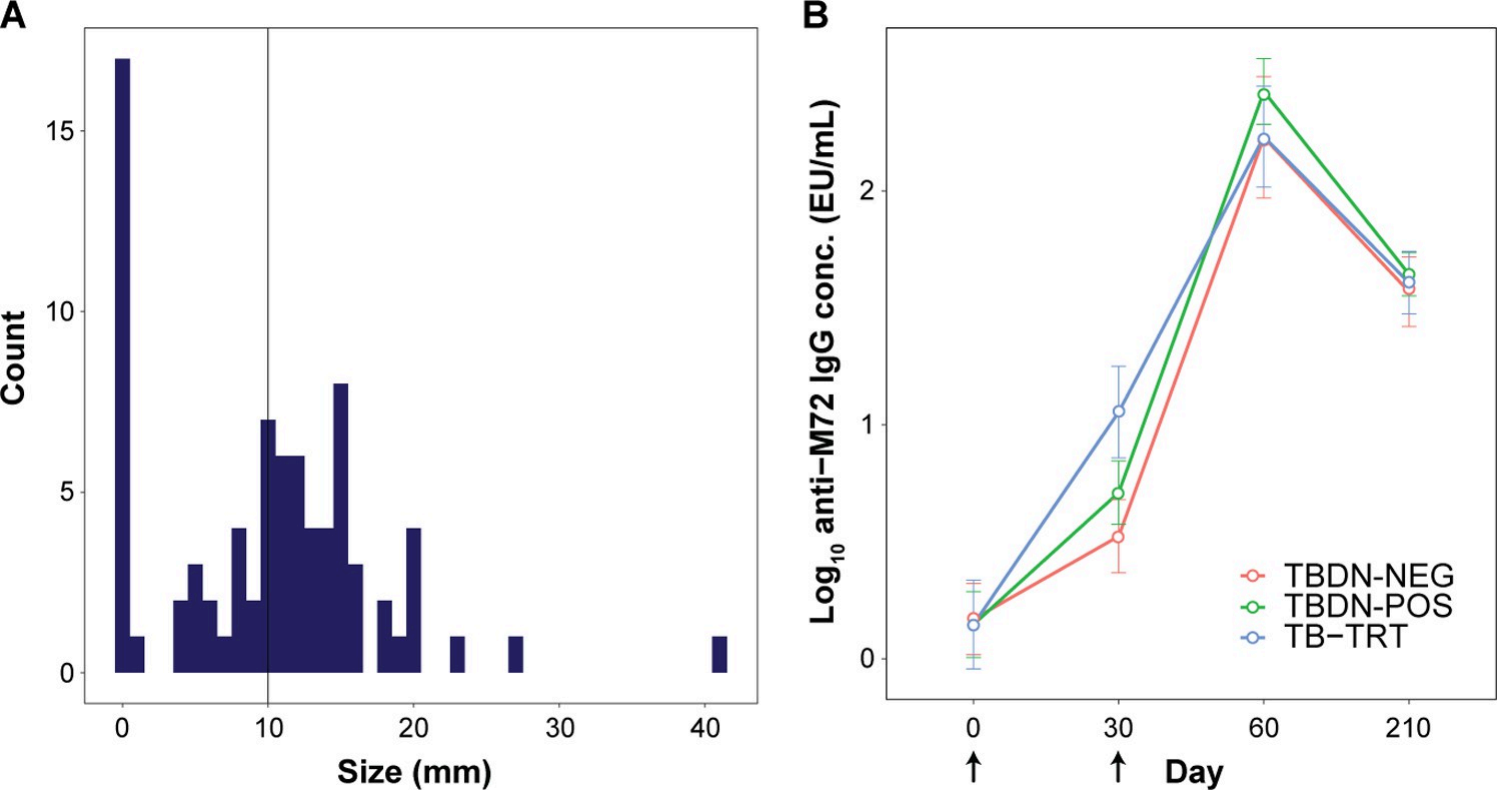
Trial design, PPD distribution and group characteristics. (**A**) Histogram presents the size of purified protein derivative (PPD) skin test indurations, as measured at baseline (Day [D]0) in the full TB disease-naïve (TBDN) cohort defined in the primary study (N = 80 [21]). Participants were considered PPD-positive using the depicted threshold (vertical line) of ≥ 10 mm. In the current analysis, M72/AS01 vaccine recipients of the TBDN cohort (N = 40) were subsequently assigned to the PPD-positive (TBDN-POS) or PPD-negative (TBDN-NEG) groups (N = 25 and 15, respectively). (**B**) Log_10_-transformed anti-M72 immunoglobulin G (IgG) antibody concentrations (means ±95% confidence intervals) are presented for the TBDN-POS, TBDN-NEG, and TB-treated (‘TB-TRT’; N = 24) groups. M72/AS01 was administered at D0 and D30, as indicated by the arrows. M72-specific antibody concentrations were measured before the first dose (D0), 1 month post-dose 1 (D30), and 1 and 6 months post-dose 2 (D60 and D210, respectively). Samples below the assay cut-off of 2.8 Elisa unit (EU)/mL were assigned a value of 1.4 EU/mL.

Analyzed subsets of the primary study participants [21] comprised TB-treated (‘TB-TRT’) and TB-disease-naïve (‘TBDN’) M72/AS01 vaccinees who were seronegative for HIV-1 and HIV-2 antibodies (N = 24 and 40, respectively; **Fig 1**). Per-protocol immunization was at Day (D)0 and D30, and all but three of these participants received two vaccine doses. Baseline skin test (PPD) data showed that although most of the 80 TBDN placebo or vaccine recipients had indurations < 20 mm, the induration size range was wide (0 to 41 mm; **Fig 2A**). Therefore, using a ≥ 10 mm cut-off [31, 33], we assigned the TBDN M72/AS01 recipients to PPD-negative (TBDN-NEG) or PPD-positive (TBDN-POS) groups (N = 15 and 25, respectively; **Fig 1**). Demographic and pre-exposure-related group characteristics are summarized in **Table 1**. The groups were overall age- and gender-balanced. However, participants in the TB-TRT group were from White-Caucasian/European or Asian–East Asian heritages, while the other two groups were composed completely of participants from Asian–East Asian heritage. This imbalance across groups was addressed in the data analysis strategy by including ethnicity as a cofactor in the linear mixed models.

**Table 1:** Group descriptions.

	N	Age (y)	Trial site	Participant heritage* (n)	Sex by ethnicity; % (n)	(Interpretation of) immune and/or pre-exposure status by group				
						BCG-primed % (n)	Immunememory	Adaptive response at the time of (putative) infection	Status at screening	Explanation
TBDN-NEG	15	20–54	Taiwan	A–EA (15)	F: 33.3 (5)	100 (15)	Absent	Not applicable	Not infected	Most likely: no natural encounter with Mtb. Possibly: TST reversion and/or elimination of an infection by innate immunity, without developing immunological memory.
					M: 66.7 (10)					
TBDN-POS	25	23–59	Taiwan	A–EA (25)	F: 44.0 (11)	100 (25)	Present	Productive (asymptomatic infection)	Not infected or LTBI	Most likely: asymptomatic natural exposure to Mtb, controlled by adaptive immunity developing into a memory response, with complete or incomplete clearance of Mtb.
					M: 56.0 (14)					
TB-TRT	24	24–59	Estonia	W-C/E (13)	F: 20.8 (5)	91.7 (22)	Present	Non-productive (symptomatic infection)	Cleared infection or LTBI	Prior symptomatic Mtb exposure led to a non-productive adaptive response, and subsequent immunological memory
					M: 33.3 (8)					
			Taiwan	A–EA (11)	F: 33.3 (8)					
					M: 12.5 (3)					

n: number of female participants in the indicated group. y: years. TBDN: tuberculosis (TB) disease-naïve, i.e. no active pulmonary disease as indicated by chest X-ray, no signs/symptoms of TB disease, and no history of chemoprophylaxis or treatment for TB. TBDN-NEG/TBDN-POS: TBDN purified protein derivative (PPD)-negative/positive, as based on the induration size (< 10 mm/≥ 10 mm) in the tuberculin skin test (TST). TB-TRT: TB-treated, i.e. with a history of successful treatment of pulmonary TB disease received at least 1 y before vaccination, with no active pulmonary disease on chest X-ray.

*A–EA, Asian–East Asian heritage. W-C/E, White-Caucasian/European heritage. BCG-primed: with documented previous Bacille Calmette-Guérin (BCG) vaccination and/or the presence of a BCG scar. Interpretation of the immune statuses was based on the collected BCG status and PPD skin test data, and the pre-exposure and disease histories [21]. Mtb: *Mycobacterium tuberculosis*. LTBI: latent tuberculosis infection.

All TBDN participants, and the vast majority of TB-TRT participants were BCG-primed. We assumed that lack of immunological memory (as defined by PPD induration < 10 mm) in TBDN-NEG participants was most likely due to lack of any contracted Mtb infections, though occurrence of prior, strictly innate immunity-controlled asymptomatic infections [34, 35] could not be excluded. Given that the TBDN-POS participants presented immunological memory and thus LTBI at screening, it was assumed that their adaptive immunity had been productive in clearing symptomatic Mtb infection [34]. While no PPD data were available for the TB-TRT cohort, presumably the immune response at the time of infection had been insufficient in controlling symptoms, but immunological memory had been developed.

Since we previously detected associations between transcriptional and antibody responses in recipients of AS01-adjuvanted vaccines [14, 17, 20], we first investigated whether different levels of pre-existing immunity translated into differential anti-M72 antibody levels. Of note, these levels are used mainly as a metric for vaccine responsiveness, as the role of humoral immunity in protection against TB remains unclear [36]. All subjects were seronegative at D0, except for one TBDN subject who had a baseline antibody level that slightly exceeded the assay cutoff (3.1 vs 2.8 EU/mL), and was classified as TBDN-NEG based on the PPD skin test results. Each vaccination induced a significant increase in levels across the initial cohorts [21]. Though the original study did not present any statistical comparison between its cohorts, levels in that study tended to be higher in the TB-TRT cohort at 1 month post-dose 1 (D30), but similar between cohorts at 1 or 6 month(s) post-dose 2 (D60 and D210, respectively) [21]. Trends in the data for the present subsets were similar to those in the original study, with a tendency of higher levels in the TB-TRT subset vs the TBDN-NEG subset at D30, but similar levels between these groups at D60 and D210 (**Fig 2B**). We used a linear mixed model to investigate the overall effect of the TB status on the antibody concentration. To account for the longitudinal nature of the data, we introduced subject identifier as a random effect. We found a significant effect of time on antibody levels (*p* < 2^−16^), as expected, while there was no significant effect of group baseline. In contrast, a significant effect of the interaction between timepoint and group on antibody profiles was observed (*p* = 0.01), suggesting that the effect of TB exposure on antibody levels varies by timepoint. Nevertheless, at D210, all participants reached similar antibody levels. Overall, this suggested that the differences in baseline immunological memory across the groups—at least to the extent that could be derived from exposure histories and PPD data—did not translate in different antibody responses. This is consistent with previous observations for this vaccine [37, 38].

### Similarity in gene expression profiles between groups is highest after the second dose

**Fig 3:**
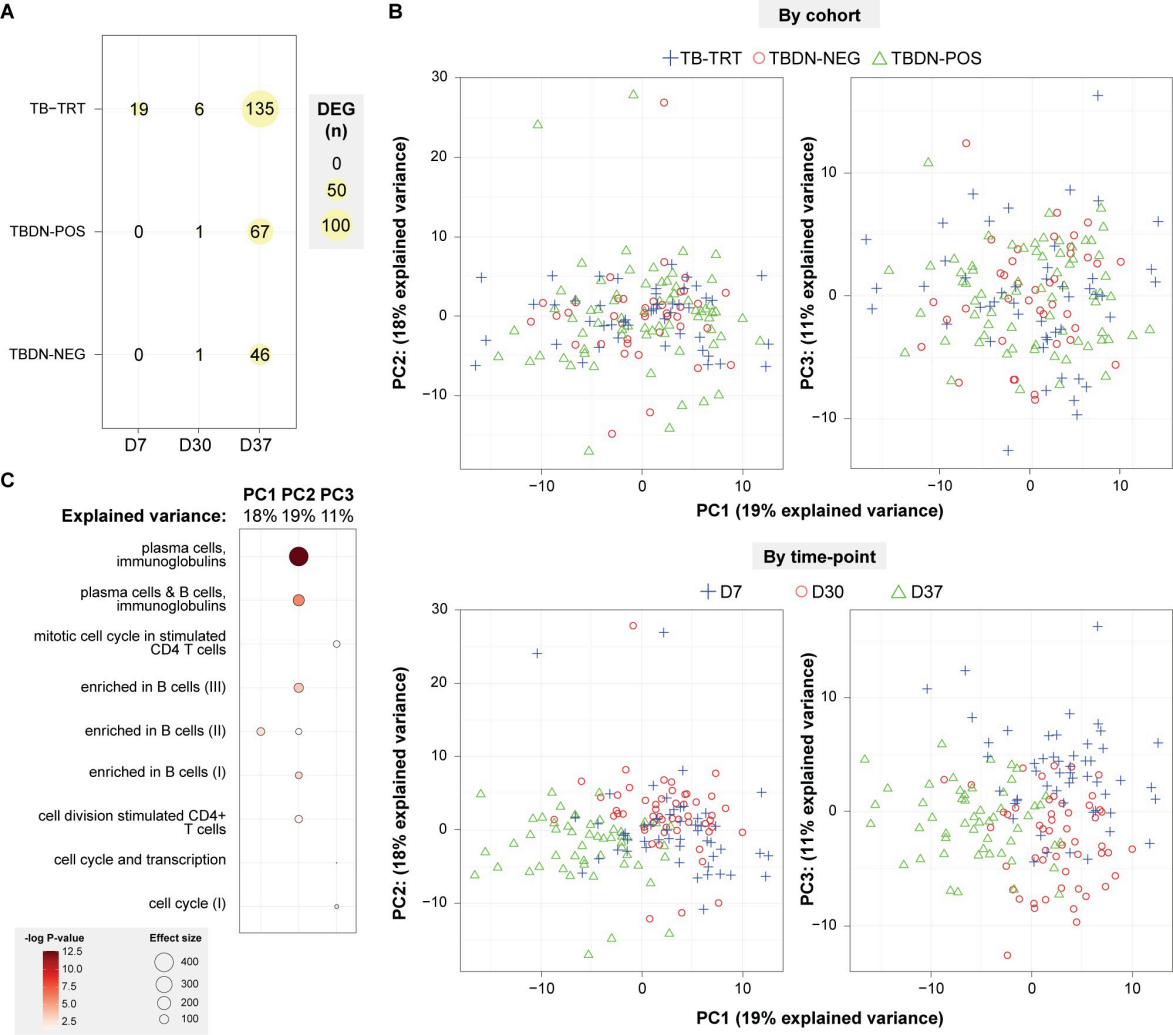
Kinetics and clustering patterns of transcriptional responses to M72/AS01 vaccine. Gene expression values in fold changes over Day (D)0 (baseline) were determined at 7 days post-dose 1 (D7), and before and 7 days after dose 2 (D30 and D37) for the tuberculosis disease-naïve (TBDN) purified protein derivative (PPD)-negative (TBDN-NEG), TBDN PPD-positive (TBDN-POS), and tuberculosis-treated (TB-TRT) groups (N = 15, 24 or 25, and 22 or 24 per timepoint, respectively). (**A**) Numbers of significant differentially expressed genes (DEG; false discovery rate [FDR] *p* < 0.05) are presented, yielding 269 DEG of the total of 1607 genes retained after a-specific filtering. Bubble size is proportional to the number of DEG, as indicated in the key. (**B**) Gene expression values were summarized by multilevel principal component (PC) analyses (PCA) of DEG fold-changes over D0, and are presented in PC1/PC2 and PC1/PC3 score plots. Plots are presented by group (top) and by timepoint post-vaccination (bottom), and color-coded according to the keys above the plots. (**C**) Bubble plot representing the blood transcriptional modules (BTMs [39]) found to be enriched (FDR *p* < 0.05) in gene-set enrichment analyses of the data by the PC1, PC2 and PC3 of the PCA data presented in (B). DC, dendritic cell.

We next assessed the impact of the level of pre-exposure to Mtb antigens on the vaccination-induced gene expression. Transcriptional responses in PBMCs collected at D7, D30, and D37 (expressed in fold-changes [FC] over D0), were analyzed using linear modeling for microarrays (limma) as described [19]. Signals were modest (20 differentially expressed genes [DEG]; TB-TRT group) or absent (other groups) at D7, and negligible (≤ 7 DEG) across groups at D30 (**Fig 3A**). All groups displayed stronger responses at D37, with the highest levels for the TB-TRT group and then the TBDN-POS and TBDN-NEG groups (133, 72 and 35 DEG, respectively), following the expected trend in CMI memory response levels between the groups based on exposure histories and PPD data. Direct statistical testing of changes in gene expression over baseline across the different participant groups did not yield any DEG except for *SPATA5* (Entrez Gene ID: 166378), the expression of which was significantly different between the TBDN-NEG and TB-TRT groups at D37. The encoded SPATA5 protein is a member of the AAA (ATPase associated with diverse activities) protein family, with no known direct or indirect effect on immunity.

Results of a qualitative analysis of gene expression at D37 over baseline across different groups are presented in **S1 Fig** and **S1 Table**. Enrichment analysis using predefined blood transcription modules (BTMs [39]) did not reveal any significant over-representation of specific functions in qualitatively different genes (data not shown). However, 67% of the genes differentially expressed in all three groups were expressed in B cells (**S2 Table**).

**Fig 4:**
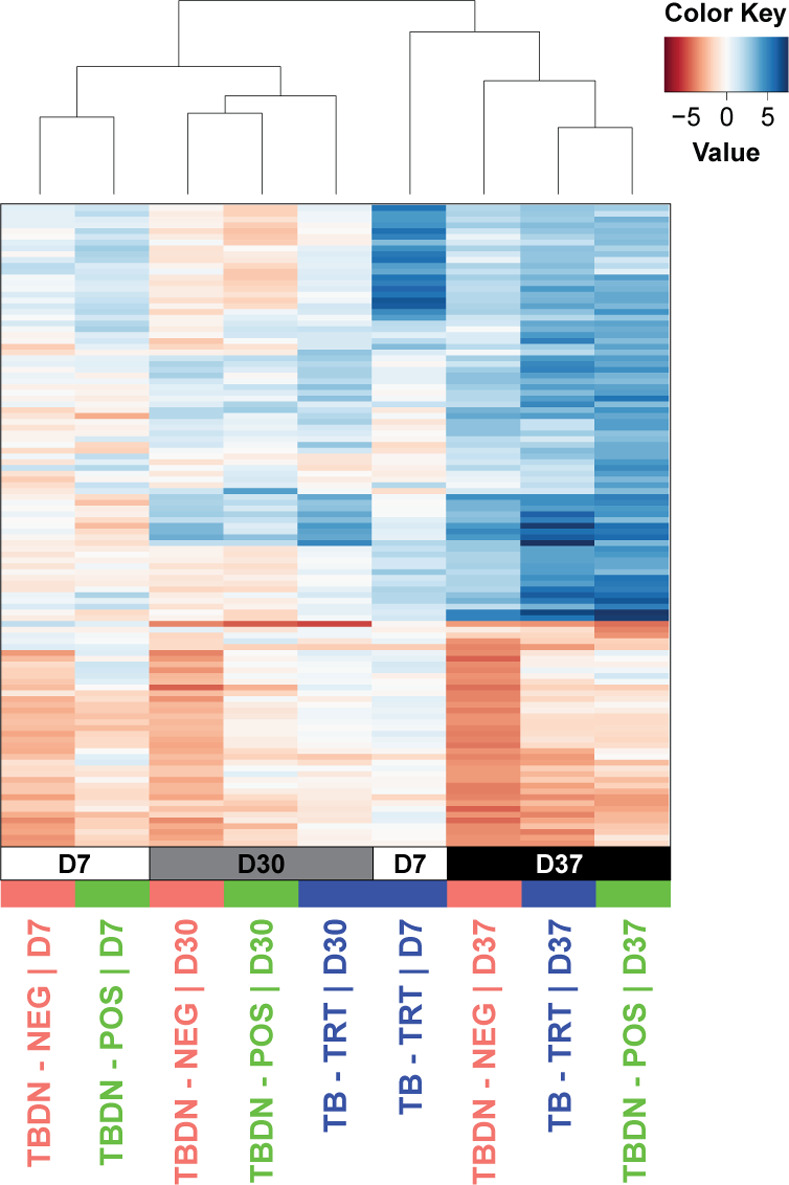
Clustering patterns of timed gene expression profiles. Heatmap representing expression values for any gene differentially expressed at any timepoint across groups and timepoints (columns, in days [D]) in positive/negative moderated t-statistics, color-coded according to the key right of the heatmap. Groups included tuberculosis disease-naïve (TBDN) purified protein derivative (PPD)-negative (TBDN-NEG), TBDN PPD-positive (TBDN-POS), or tuberculosis-treated (TB-TRT) participants (N = 15, 24 or 25, and 22 or 24 per timepoint, respectively). The dendrogram above the heatmap represents the global similarities between the timed gene expression profiles per group, as obtained by hierarchical clustering of the timepoints per group according to the overall transcriptional profile (all differentially expressed genes [DEG] at any timepoint).

Further investigation using multilevel principal component (PC) analysis (PCA) of the by-subject values confirmed that transcriptional responses varied by timepoint, as evidenced by the separation of D37 data along the PC1 axis, but did not vary clearly by group (**Fig 3B**; *left and right respectively*). Subsequent gene-set enrichment analyses of the data in each of the first three PCs was performed as previously described [19] to identify significant associations (false discovery rate [FDR] *q* < 0.05) between transcriptional response patterns and predefined BTMs (described in [39]). The data revealed enrichments in PC1 and PC2, pertaining mostly to genes in the B-cell and plasma-cell related BTMs, while only minor or no enrichment was seen in the PC3 (**Fig 3C**). To further dissect the results, we performed hierarchical clustering of the timed group data, and observed that clustering patterns were generally based on timepoints rather than on groups (**Fig 4**), consistent with **Fig 3B**. No marked effect of PPD status was seen in the D7 profiles of the two TBDN subsets, which both clustered with the D30 profiles generated for all three groups. While post-dose-1 (D7, D30) and post-dose-2 (D37) profiles were typically clearly separated, the D7 profile of the TB-TRT group clustered with the joint D37 profiles of all three groups. This suggests that the disease-experienced individuals exhibited, already after the first vaccination, a transcriptional response which resembled the recall response observed in the disease-naïve individuals. The effect was mediated by differences in the levels of CMI memory responses displayed by these participant groups.

### Accelerated cell function and proliferation-associated response in TB-treated individuals

**Fig 5:**
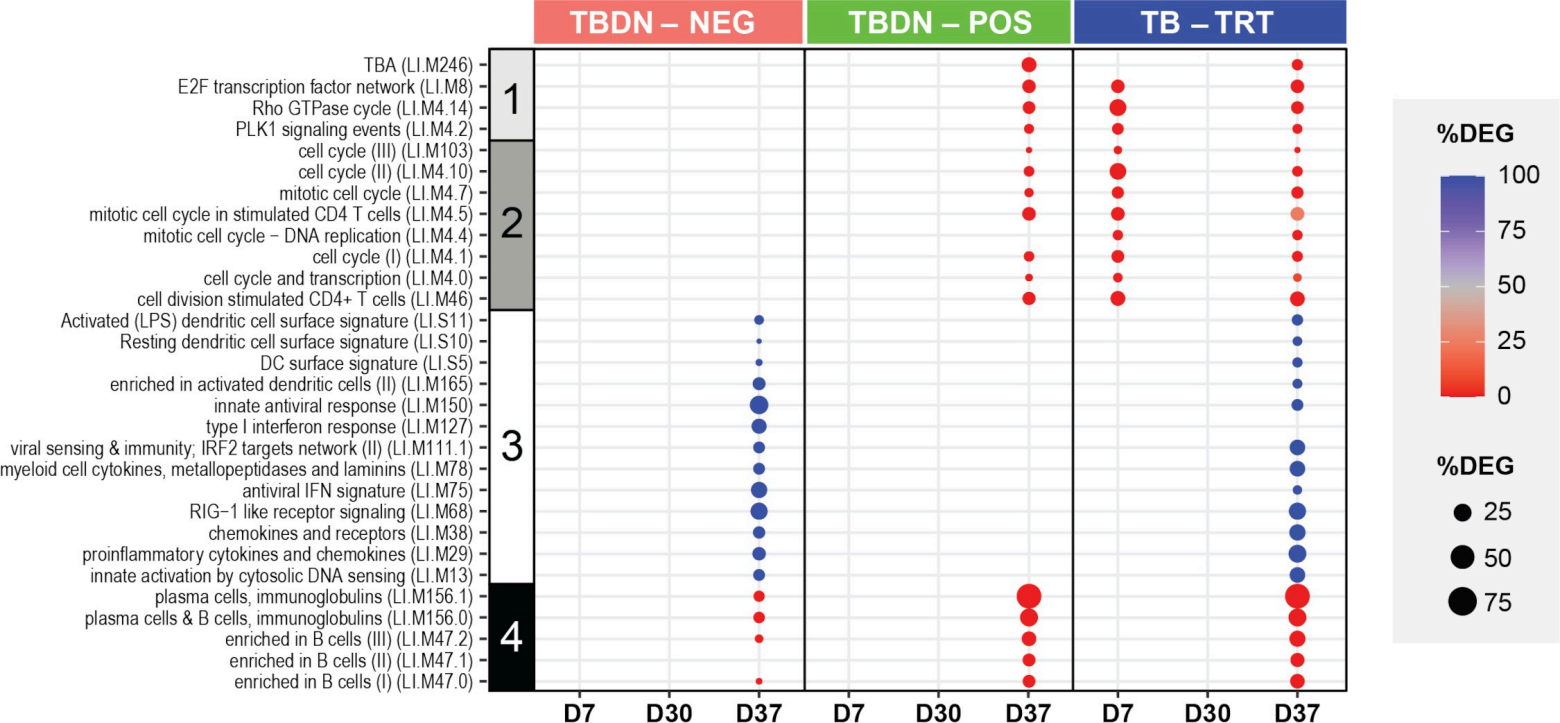
Functional characterization of the gene expression by study group. Activation levels in the 30 blood transcriptional modules (BTMs [39]) found to be enriched (false discovery rate [FDR] *p* < 0.05) by a hypergeometric test are presented by group of subjects and by timepoint (with a listing of the genes underlying these enrichments presented in **S3 Table**). Groups included tuberculosis disease-naïve (TBDN) purified protein derivative (PPD)-negative (TBDN-NEG), TBDN PPD-positive (TBDN-POS), and tuberculosis-treated (TB-TRT) subjects. As indicated by the numbers 1–4 at the left side of the plot, modules were, according to their function, assigned to BTM groups related to general cellular (signaling) mechanisms (1), cell proliferation (2), innate immune mechanisms (3), or adaptive immune mechanisms (4). As indicated by the figure keys, sphere color indicates the percentage of upregulated (blue) or downregulated (red) differentially expressed genes (DEG) among all DEG in the BTM; sphere size is proportional to the percentage of DEG in the BTM (i.e., number of DEG [FDR *p* < 0.05] among all genes in the BTM × 100, over the number of genes in the given BTM present on the microarray chip).

To facilitate interpretation of any functional differences in gene expression between timepoints and participant groups, we then performed gene-set enrichment analyses as described for **Fig 3**. Thirty BTMs were identified as being significantly enriched (**Fig 5**). They contained genes related to either general cellular mechanisms, cell-proliferation regulation, innate mechanisms, or plasma-cell and B-cell functions (4, 8, 13 or 5 BTMs, nos. 1–4 in **Fig 5**, respectively; see **S3 Table** for individual genes). Most of the innate-immunity-related BTMs (i.e., LI.M127, LI.M75, LI.M150 and LI.M68) were dominated by IFN-inducible genes. Of note, we here classified three BTMs annotated as regulating CD4^+^ T-cell cycling (LI.M46, LI.M4.5 and LI.M4.6) among the modules regulating cell proliferation. This was done because gene ontology analysis (http://geneontology.org), performed to identify enriched pathways of functionally-related gene groups, demonstrated that the genes in these BTMs were mainly involved in general cell-cycle processes, without clear attribution to a specific cell type.

Aligned with the group’s accelerated and stronger transcriptional response (**Fig 3**), only the TB-TRT subset displayed enrichments at D7, comprising upregulated responses for nearly all (4/5) general cellular-mechanism-related BTMs and all cell-proliferation-related BTMs. The minor DEG responses at D30 (**Fig 3A**) did not enrich any BTM. As expected, most enrichments were seen at D37 (9, 18 and 25 BTMs in the TBDN-NEG, TBDN-POS and TB-TRT groups, respectively). At this timepoint, general cellular-mechanism-related modules were only enriched in the TBDN-POS and TB-TRT groups (5/5 BTMs upregulated). By contrast, innate immunity-related BTMs were only enriched in the TBDN-NEG group, and, at lower DEG frequencies (‘intensities’; see key in **Fig 3**), in the TB-TRT group (7/7 and 6/7 downregulated BTMs, respectively). Across the groups, nearly all (TBDN-NEG group; 4/5 BTMs) or all (other groups) B-cell or plasma cell-associated BTMs were enriched at D37, and all were upregulated. However, due to just-below-threshold values for many of the associated genes, intensities in the TBDN-NEG group were lower than in the other groups (≤ 20% vs ≤ 75%, respectively). Finally, the most prominent signals in terms of enriched BTMs were the uniformly upregulated cell-proliferation-related modules detected only for the TBDN-POS and TB-TRT groups (8/9 BTMs each), though most of these signals were of modest (≤25%) intensity. Overall, the nature and kinetic patterns were consistent with time-matched data previously generated for this and other AS01-adjuvanted vaccine(s) [19, 20].

### Potential link between D37 B-cell signals and antibody responses

We next investigated whether the B-cell signals at D37 were associated with the subsequent anti-M72 antibody concentrations. To that aim, we first summarized, for each group, the D37 expression values of genes belonging to the five identified B-cell modules, using PCA. Score plots of the PC1 and PC2 (corresponding to 50% and 22% of the explained variance, respectively) revealed no clear separation between groups along the PC1 axis, though all of the highest PC1 scores belonged to Mtb-exposed individuals (**S2A Fig**). When we plotted the PC1 scores against the antibody levels at D60 and D210 (**S2B** and **S2C Fig**, respectively), we observed a possible positive association between these parameters across each of the three groups, particularly for D210. This was confirmed by the finding that D37 PC1 scores had a statistically significant effect, as detected when we used a linear mixed model to assess the effects of time (*p* < 2×10^−16^) and the PC1 (*p* = 0.03686) on anti-M72 antibody levels. Timepoint by timepoint analysis showed a stronger association between PC1 and the antibody levels at D210 vs D60, without significant group effect (*p* time effect: 0.09939 [D60] and 0.01646 [D210]).

To interrogate the data underlying the differential BTM enrichment levels between the groups (**Fig 4**) at the gene-level, we generated heatmaps of the expression values (log_2_ FC over D0) of the associated genes (**S3 Fig**). The plots revealed that the apparent inter-group differences were mainly attributable to variations in sample size and intensity of gene perturbation, rather than to distinct patterns. For example, expression values of the 23 genes underlying the upregulated cell proliferation-related modules, revealed that the upregulated D7 signals were not restricted to the TB-TRT group but were, at lower intensities, also detected in the other groups. Except for *RIN2* and *LAG3*, all these genes were most prominently perturbed at D7 in the TB-TRT group, at values that were higher than the D37 values for the same group, or than the D7 or D37 values for both other groups.

The nature of the transcriptomic responses was thus overall similar between groups, even though early proliferation-associated signals were more prominently displayed by TB-treated individuals. This prompted the hypothesis that these signals could cue the induction of adaptive immunity.

### D7 proliferation signal is linked to humoral responses, regardless of pre-existing immunity to TB

**Fig 6:**
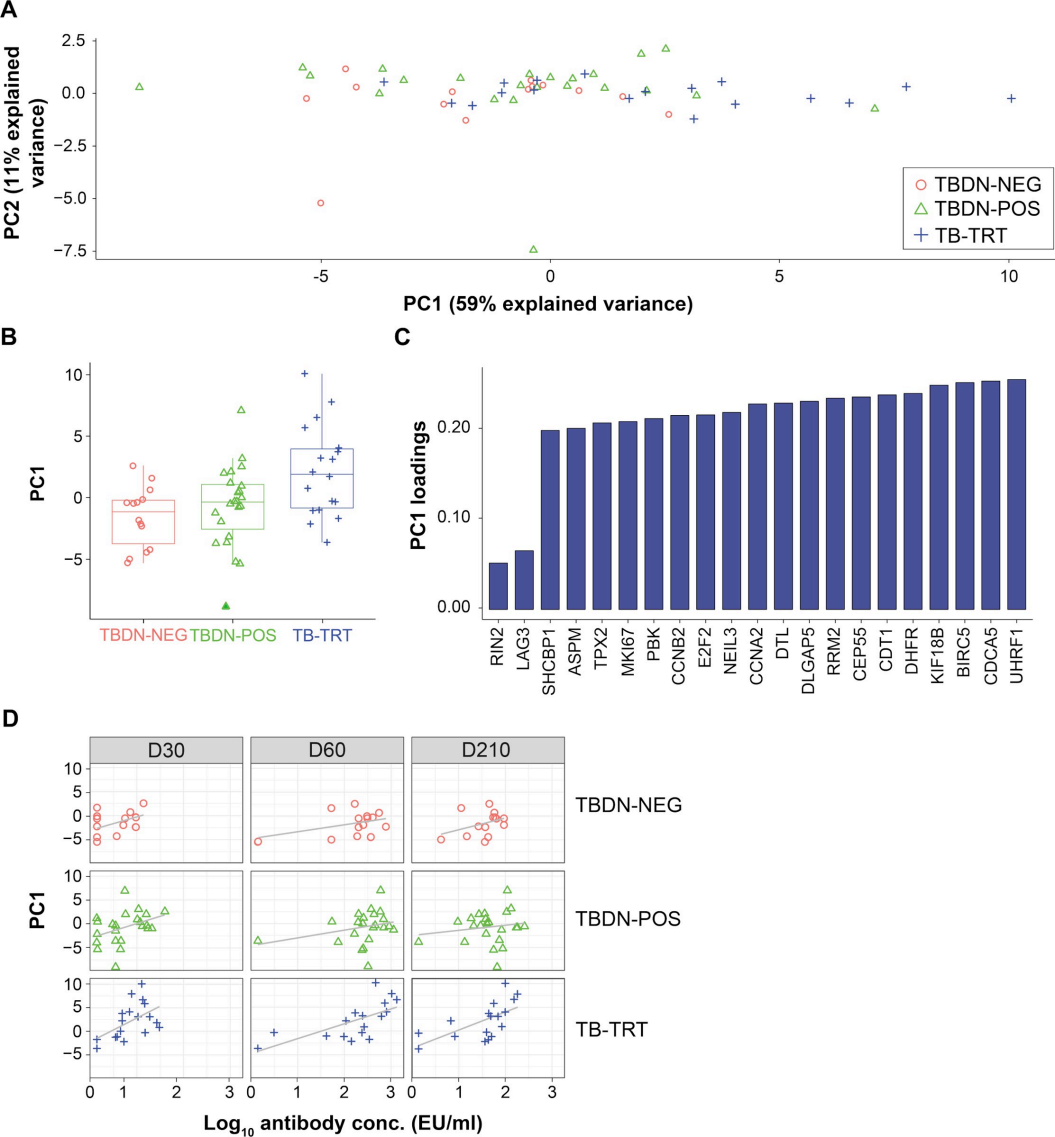
Day 7 proliferation signals are associated with the antibody response. Principal component (PC) analysis was performed on the fold-change expression values (Day [D]7 vs D0) of the 23 genes in the blood transcriptional models (BTMs) associated with cell proliferation (see **Figs 5 and S3**). Data are represented for the tuberculosis (TB)-disease-naïve (TBDN), purified protein derivate (PPD)-negative or -positive (TBDN-NEG or TBDN-POS, respectively) and TB-treated (TB-TRT) groups. Each symbol in panels A, B and D represents an individual subject. (**A**) The biplot presents the scores in the first two PCs (PC1 and PC2, respectively). (**B**) Boxplots represent the distribution (median, interquartile range, minima, and maxima) of PC1 scores by group. (**C**) PC1 scores of the 23 genes in the proliferation-associated BTMs are shown for the TB-TRT group. (**D**) Relationship between scores in PC1 and the log_10_ anti-M72 antibody concentrations (expressed in Elisa Units [EU]/mL) measured at D30, D60 and D210 are shown by group. Grey lines representing indicative trends without statistical connotation were added to facilitate interpretation.

To test the hypothetical relationship between the early cell proliferation signals and subsequent humoral responses, we first summarized the by-subject expression values (FC D7/D0) of the 23 implicated genes by PCA. The lack of a distinct intergroup separation in the scores of the PC1 and PC2 (representing 59% and 11% of the explained variance, respectively; **Fig 6A**) suggested that the proliferation signals were relevant for each group, even though all highest PC1 scores were mostly exhibited by TB-TRT participants. This was confirmed by the distribution of PC1 scores, showing slightly higher medians for the latter individuals (**Fig 6B**), and by the higher PC1 scores for most (21/23) of the genes displayed by this group (**Fig 6C**). To test the association with the adaptive response, we then plotted anti-M72 antibody concentrations measured at D30, D60 or D210 against the individual PC1 scores by group and timepoint (**Fig 6D**). While most visible in the TB-TRT group, the regression line slopes suggested the presence of positive associations for all three groups. Linear modeling of the antibody data, as function of either the timepoint or the PC1 score, confirmed a significant effect of the timepoint (*p* < 2×10^−16^) and PC1 (*p* = 0.0040) on antibody levels. The statistical significance of the effect was confirmed by similar modelling performed for each antibody timepoint (*p* = 0.0171, *p* = 0.00017 and *p* = 0.001752, at D30, D60 and D120 respectively; without significant group effect). This result was aligned with the similar distribution of subjects between the groups, as observed when we stratified the individual antibody levels by PC1 score category (**S4 Fig**).

Altogether, the proliferation signals at D7 were in all participant groups associated with the magnitudes of antibody responses detected either 1 month post-dose 1 or 7 days post-dose 2. Although this association was thus observed irrespective of the host’s level of baseline CMI memory responses, it was strongest in the individuals with presumably the most robust immunological memory.

#### Immunological memory affected response heterogeneity after the first, but not the second dose

Group average-based gene expression analyses can conceal differences in the level of interindividual diversity of immune responses between separate populations [17]. Given the largely overlapping gene expression patterns seen thus far for the three groups—and considering that a second dose of a different AS01-adjuvanted vaccine can moderate the interindividual response heterogeneity seen after the first dose in baseline-naïve subjects [17]—we next investigated whether the presence of high-level immune memory would give rise to more homogeneous within-group responses to the vaccine antigens.

**Fig 7:**
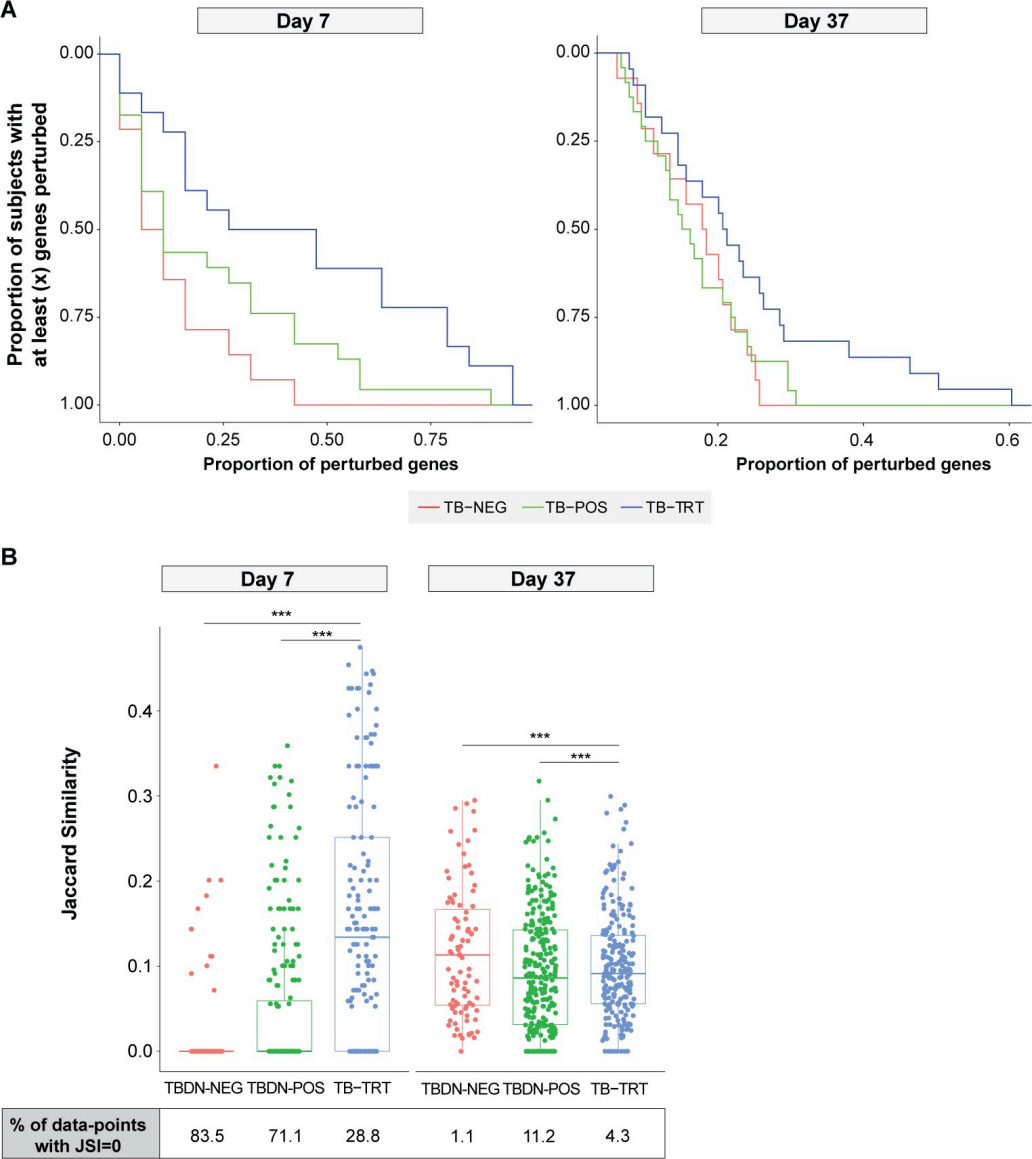
Tuberculosis exposure results in different heterogeneity of the response, which levels after repeated immunization. Heterogeneity analyses were performed on all differentially expressed genes (DEG) observed in at least one timepoint and one condition and are presented for D7 and D37 (7 days following the first or second vaccination, respectively). Participant groups included M72/AS01 vaccinees who were either tuberculosis disease-naïve (TBDN) purified protein derivative (PPD)-negative (TBDN-NEG), TBDN PPD-positive (TBDN-POS), or tuberculosis-treated (TB-TRT); total number of vaccinees = 55 and 60 at D7 and D37, respectively. (**A**) Reverse cumulative distribution plots show the interindividual heterogeneity in gene expression (in numbers of responsive genes) by timepoint and participant group (**B**) Boxplots compare the distribution of Jaccard similarity index (JSI) data of pairs of different group × timepoint conditions, with percentages of datapoints with a JSI value of 0 indicated by group and timepoint below the plot. Bars represent the medians, and first and third quartiles; lower/upper whiskers represent the lowest/highest value within 1.5 × the interquartile range. Asterisks above the plots denote statistical significance in Student’s t-tests when comparing the means (***p < 0.001). Proportions of subjects with completely distinct gene expression profiles (i.e., JSI = 0) are indicated by group and timepoint below the graph.

We first compared the response heterogeneity between post-dose 1 (D7) and post-dose 2 (D37) responses, using reverse cumulative distribution analysis of the gene expression by participant group and by post-vaccination timepoint (**Fig 7A**). Overall, we observed a high level of heterogeneity in the response at both timepoints. Yet, within all three groups, responses at D7 were more heterogeneous as compared to D37. These trends were further illustrated by heatmap analysis of the individual (by-subject) expression values (FC vs D0) for all DEG identified at these timepoints, while excluding the negligible D30 responses (**S5 Fig**).

We then refined the quantification of within-group response heterogeneity, using Jaccard similarity index (JSI) data computed between all possible pairs of subjects of the respective group (**Fig 7B**). As JSI values are proportional to the fraction of DEG shared between two subjects (ranging from 0 [no overlap or homogeneity] to 1 [maximum overlap or homogeneity]), the obtained JSI distributions revealed that the homogeneity levels were proportional to the levels of pre-existing immunity (TB-TRT > TBDN-POS > TBDN-NEG) after the first dose, but comparable between the groups after the second dose. Similarly, the proportions of subjects with completely distinct gene expression profiles (i.e., JSI = 0) after the first and second vaccination could differ substantially between the groups (see **Fig 7B**). this trend was also supported by linear modelling (unbalanced ANOVA) of JSI values, demonstrating that not only the parameters ‘group’ and ‘timepoint’, but also their interaction (representing the group response patterns), significantly affected the homogeneity levels (*p* < 2×10^−16^, *p* = 1.3×10^−8^, and *p* < 2×10^−16^, respectively).

Altogether, this indicates that low-level immunological memory at baseline promoted an increased response heterogeneity upon the first immunization, an effect that is negated by a second immunization, which then equalized the homogeneity levels across the strata of immunological memory of the vaccinees.

## Discussion

The paucity of data describing the effect of pre-existing immunological memory, or diversity therein, on functional transcriptional responses to adjuvanted vaccines, can limit optimal design of vaccine trials in different populations. In this post-hoc analysis of clinical trial samples, we compared the molecular signatures elicited by two immunizations with a relevant AS01-adjuvanted vaccine, between three groups of adults with variable levels of pre-exposure and baseline immune memory. Key observations deduced from these analyses comprised that (i) signatures were not distinctly different between the groups; (ii) the disease-treated individuals displayed a stronger proliferation-related signal upon the first dose as compared to the less-exposed individuals, and across the groups these early signals were associated with an increase in antibody responses after each immunization; (iii) having a history of TB resulted in more homogenous gene expression between individuals after the first dose, but this effect was leveled after a second dose, when the response homogeneity was similar between the three groups.

Overall, the transcriptional responses to M72/AS01 vaccination observed here in antigen-experienced individuals are well aligned with previous reports describing the responses to this or other AS01-adjuvanted vaccines in naïve recipients [17, 19, 20]. In particular, the post-peak B-cell and proliferation-related signatures seen at 1 week post-dose 2 (D37) in each study group (though most prominently in both infected groups) overlapped with previous time-matched observations in naïve recipients of either this vaccine, RTS,S/AS01 or HBs/AS01. Such signatures were also detected 7 days after a single dose of influenza or yellow fever (YF-17D) vaccine, in primed or naïve individuals respectively, though for the latter (live-attenuated) vaccine this represented the peak response [40–42]. In addition, the currently observed D7/D37 proliferation signals were also detected in naïve RTS,S/AS01 recipients [20]. Although we have no data to support that these D7/D37 proliferation signals were driven by plasmablasts, activated plasmablasts were in the cited transcriptional studies reported at D7 [20] and/or D37 [17, 19, 20]. This suggests a potential link between this cell type and the proliferation signals reported here. However, differences with published data were also identified, such as the clearly downregulated innate and IFN signatures seen at D37 in the naïve group. Indeed, equivalent modules were previously found to be *up*regulated in time-matched signatures from naïve M72/AS01 or RTS,S/AS01 recipients [19, 20]. The disparity in M72/AS01 data might be due to the sample type (purified PBMC here, vs the aggregate of cell types in WB analyzed previously), while differences with RTS,S/AS01 data may be due to the adjuvant dose (AS01_E_ for M72 vaccine, vs AS01_B_ [containing twice the MPL and QS-21 quantities of AS01_E_] in the cited RTS,S study). The latter assumption on adjuvant dose is supported by recent WB-derived data comparing different HBs vaccines in naïve subjects, showing more numerous and mostly (25/32) downregulated innate- or IFN-related BTMs at D37 for a formulation with AS01_B_, and fewer and universally (13/13) upregulated BTMs with AS01_E_ [17]. The current lack of NK-cell signatures was another notable difference with published data for other AS01-adjuvanted vaccines, which demonstrated NK enrichments persisting at D37 (following downregulated responses 1 day post-vaccination) [17, 19, 20].

Though the lack of a non-adjuvanted M72 vaccine group precluded full discrimination of strictly AS01-mediated effects, the gene expression patterns identified in the current study were consistent with those induced by other AS01-adjuvanted vaccines, indicating that a generic gene expression signature for AS01-adjuvanted vaccines may exist. Most notably, this gene expression signature identified the B-cell responses induced by AS01-adjuvanted vaccines [19].

The more robust signals in the disease-treated individuals, seen both in early (D7) proliferation-related modules and in subsequent overall gene expression, suggested that strong cell-mediated immunological memory, such as generated after an uncontrolled infection, stimulated and accelerated the transcriptional response to vaccination. Likewise, for other AS01-adjuvanted vaccines we found that gene expression for overlapping or equivalent proliferation modules in naïve individuals was undetectable or negligible 3–6 h (HBs) or 1 day (RTS,S) after the first dose, but could be detected at corresponding timepoints after the second dose, with typically higher intensities at subsequent timepoints after the second vs after the first dose [17, 20].

Interestingly, an association between molecular signatures—here, D7 proliferation-related or D37 B-cell-related signatures—and the magnitudes of subsequent antibody responses, was also observed with other vaccines. For example, such associations were seen in HBs/AS01, H5N1/AS03 and seasonal influenza vaccinees, involving mainly IFN-related signals, as well as in RTS,S/AS01 and YF-17D vaccinees, involving distinct B cell-related signals [17, 20, 40, 43, 44]. However, translation of the trend in gene expression intensity seen here (TB-TRT > TBDN-POS > TBDN-NEG), into the antibody response magnitudes was only ambiguous after the first dose (for D7 proliferation signals), and absent after the second dose (for D37 proliferation and/or B-cell signals). Apparently, infection-induced memory responses did not affect baseline or post-vaccination antibody levels, consistent with previous observations in M72/AS01 recipients. Indeed, similar antibody levels between infected and uninfected subjects were previously reported at pre-vaccination baseline for adolescent and adult vaccinees, as well as after vaccination, for adolescent vaccinees [37, 38].

Relative to the disease-treated individuals, both disease-naïve populations displayed lower-level gene expression after the first dose. The latter two groups likely also had weaker Mtb-specific CMI memory responses at baseline as compared to the disease-treated individuals, due to less pre-exposure (as supported by the TST results for TBDN-NEG participants). The lower-level gene expression in disease-naïve participants may therefore be aligned with data showing that memory CD4^+^ T cells can stimulate innate responses [17, 45]. In addition, the relatively high level of interindividual heterogeneity in the transcriptional responses after the first dose in the disease-naïve individuals may be explained by this weaker innate stimulation, which was apparently unable to fully moderate any pre-existing immunological diversity among these vaccinees. The latter was also observed after a single dose of either seasonal influenza vaccine or alum-adjuvanted HBs vaccine [3, 17, 42, 46]. These inter-group differences were abrogated after the second vaccination, which increased the response homogeneity in all groups. Similar changes in inter-individual variability in gene expression between successive doses have been reported for HBs/AS01 in naïve adults [17]. The more homogeneous response in the disease-treated individuals did not appear to translate into higher mean antibody responses. However, such translation might still be observed for the vaccine-induced T-cell response, which would be encouraging even in the absence of an immune correlate of protection. Indeed, as compared to TB-naïve subjects, disease-treated individuals can have a higher incidence of recurring TB disease, either due to endogenous reactivation of the initial infecting strain, reinfection with a different strain, and/or genetic background [47].

Finally, in the setting of this complex, dynamic disease with its continuum of manifestations, no definitive participant segregation based on strata of pre-exposure levels could be made. This is illustrated by the case of incipient TB disease, during which active disease can remain asymptomatic and without radiographic abnormalities, which could have caused erroneous assignment of participants to the TBDN cohort. In addition, spontaneous reversion from a PPD-positive to PPD-negative status, possibly provoked by trained innate immunity that successfully eradicated the Mtb infection [35, 48, 49], could have taken place at any time during the study. PPD reversion is often multifactorial (i.e., depending on BCG status, the time-span elapsed since infection, and/or exposure to nontuberculous mycobacteria cross-reacting with PPD antigens [50, 51]), therefore its occurrence is unpredictable. These uncertainties may be compounded by the relatively low specificity of the TST, at least as compared to IFN-γ release assays [31, 52], though contrasting data exist [32, 53]. In addition, the unexpected finding that the kinetics of the transcriptional response to M72/AS01 vaccination depended on the baseline Mtb exposure status, may have limited our comparison of response qualities between the groups at D7/D37. Nonetheless, while these factors may have complicated the interpretation of results, we argue that our main conclusions are maintained, particularly for the most distinct differences between the opposites in the current spectrum, i.e., TBDN-NEG and TB-TRT individuals. This study of transcriptional responses driven by AS01 also has considerable strengths, as it allows linking with the referenced published studies, and contributes to the ever-increasing datasets generated for this clinically relevant Adjuvant System.

Interpretation of the study results was limited by the small sample sizes of the study groups. This precluded correction of the data for putative immune determinants other than the priming status (identified elsewhere [1–4]), and limited the statistical power of our study overall. Due to this, and because we could not establish the stability of the transcriptional features at D0 (for instance by having access to multiple pre-vaccination samples), we were not able to assess the effect of baseline transcriptional statuses on the vaccine responses. Additionally, there was substantial variation in ethnicity across groups, although this factor was taken into account and mitigated in the data analysis strategy. Furthermore, conclusions on functional differences (**Fig 4**; BTM regulation) between groups were drawn from numbers of perturbed genes within the BTMs, and that analysis did therefore not reflect any intergroup differences in expression intensity (i.e., difference in FC values). Collectively these unknowns prevented us from providing causative explanations of the identified trends in immune responses.

## Conclusions

Pre-existing immunity at baseline accelerated and amplified the transcriptional response to a single dose of AS01-adjuvanted vaccine, resulting in gene expression levels that were more homogenous between individuals with similar infection and disease histories. However, irrespective of the level of baseline immune memory, the heterogeneity in transcriptional responses was reduced after a second vaccine dose, and attained comparable levels between groups of individuals with different degrees of pre-exposure to the pathogen.

**Fig 8:**
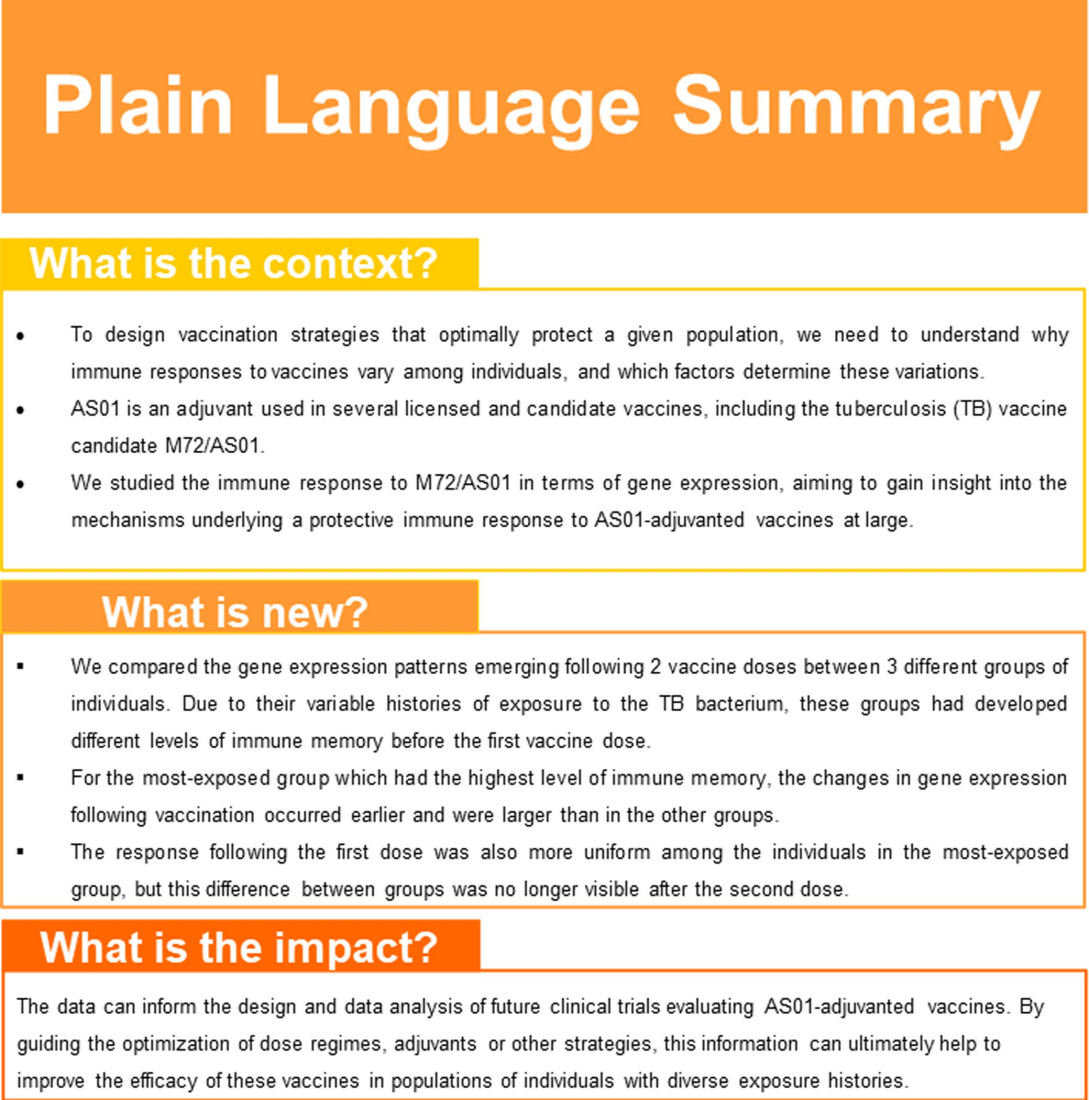
Plain language summary.

Although further research is needed, the data could be used to guide any future post-hoc analyses of bio-banked samples collected in an independent sub-study (NCT02097095) conducted in parallel with the Phase IIb efficacy trial of M72/AS01 [13]. Ultimately, the identification of mechanisms regulating responsiveness to vaccine regimens, or of a biosignature reflecting a protective response that can be detected shortly after vaccination, could facilitate future trials by reducing required sample sizes and follow-up times. In addition, this could help optimizing specific interventions (e.g., formulation, dosing, or booster regimen) for a given population. The clinical relevance of this study and its impact on the patient population are summarized in **Fig 8**.

## Materials and methods

### Ethical statement

Samples were sourced from the observer-blind, randomized, controlled Phase II trial (NCT01424501) which aimed to compare safety, reactogenicity and immunogenicity of M72/AS01 in populations with different exposure to TB at baseline [21]. The protocol was approved by all institutional Ethics Committees and conducted in accordance with the Declaration of Helsinki and Good Clinical Practice guidelines. A summary of the protocol is available at http://www.gsk-clinicalstudyregister.com (GSK study ID 114886). Written informed consent for the primary analysis, as well as for the post-hoc analysis described here, was obtained from each participant before trial participation.

### Study design and participants

Participants were healthy male or non-pregnant female adults aged 18–59 years, who were seronegative for HIV-1 and HIV-2 antibodies and living in TB-endemic countries (Taiwan and Estonia) [21]. Cohort assignment and exclusion criteria were applied in the primary analysis as described [21]. Briefly, participants with previous history of successfully treated pulmonary TB disease at least one year prior to vaccination, and with no active pulmonary disease on chest X-ray were assigned to the TB-TRT cohort, and participants who had no active pulmonary disease on chest X-ray, no clinical signs or symptoms of TB disease, and no history of chemoprophylaxis or treatment for TB disease were assigned to the TBDN cohort (**Fig 1**). A third cohort, including participants who had completed the intensive phase of TB treatment, was excluded from the current analysis.

TSTs were performed in the TBDN cohort at least 2 weeks before vaccination as described [21], and per applicable US Centers for Disease Control and Preventions recommendations [31]. TST positivity was defined as having a ≥ 10 mm induration. The current post-hoc analysis included all M72/AS01 vaccinees of the TBDN cohort stratified by PPD status into the TBDN-POS and TBDN-NEG groups, as well as all vaccinees of the TB-TRT cohort except for one subject. This individual reported a possibly vaccine-related serious adverse event (grade 3 hypersensitivity) and was thus excluded from the current analyses, as well as all participants of the groups of the original study cohorts receiving the physiological saline placebo (**Fig 1**).

Participants in the vaccine arms received M72/AS01 by intramuscular injection at D0 and D30, and all were followed until six months post-dose 2 (D210). The candidate vaccine M72/AS01_E_ (GSK, Rixensart, Belgium; referred to as M72/AS01 elsewhere in this manuscript) contains the M72 antigen (10 μg/dose) and the AS01_E_ Adjuvant System. M72 is a recombinant fusion protein derived from the Mtb32A and Mtb39A proteins [54] which are both expressed in BCG and present in PPD [55, 56]. AS01_E_ contains 25 μg MPL (produced by GSK), 25 μg QS-21 (*Quillaja saponaria* Molina, fraction 21; licensed by GSK from Antigenics LLC, a wholly owned subsidiary of Agenus Inc., a DE, USA corporation) and liposomes per dose. The current endpoint was the profiling of RNA expression by transcriptome microarrays, as described below, using PBMC samples obtained from these participants at D0, D7, D30 and D37. Anti-M72 IgG antibody concentrations were previously quantified by ELISA (cutoff ≥ 2.8 mIU/mL) at D0, D30, D60 and D210 [21], and associated descriptive statistics were performed using SAS (SAS Institute Inc., NC, USA).

### RNA expression profiling

Total mRNA was isolated from frozen PBMC samples using a standard Qiagen kit. RNA was amplified using the Ovation kit and protocol (NuGEN, CA, USA) and RNA expression levels were determined using the Human Genome-U133 Plus 2.0 arrays of 54120 probe-sets derived from gene transcripts (Affymetrix, OH, USA).

### Microarray preprocessing

RNA was extracted from isolated PBMC using standard protocols, and RNA expression levels were determined using Affymetrix HG-U133 Plus 2.0 arrays. RNA quality control (QC) included quantification and quality analysis using the Agilent 2100 BioAnalyzer (BA). The RNA concentration was measured using the RiboGreen fluorescence assay. Raw microarray data were quality-controlled using standard methods, and normalized by GC Robust MultiArray Average (GCRMA [57]) as described [19, 58]. Microarray QC included analysis of the scale factor, the percentage present, the GAPDH (glyceraldehyde-3-phosphate dehydrogenase) 3’ to 5’ ratio, the relative log expression (RLE) and the normalized unscaled standard errors (NUSE). One microarray was excluded for failing QC-criteria. After normalization, probe-sets were filtered and retained based on the interquartile range (>0.75) of RNA expression data. In addition, probe-sets referring to the same gene were collapsed to the average gene expression, and probe-sets not mapping to any known gene were eliminated. A total of 2932 of the 54675 probe-sets present on the chip (5.36%) was retained for analysis, interrogating 1607 unique gene symbols. The dataset is accessible at GSE197408.

### Gene expression modelling and BTM enrichment analysis

For this analysis, only the probe-sets with annotated gene names were included. For each sample, and for genes that were represented by more than one probe-set, the average gene expression for all probe-sets was used for the given gene. A linear mixed model (limma, R package [59]) was fitted to the RNA expression data. For each study group and each gene, moderated t-statistics were calculated for the gene’s expression at each post-vaccination timepoint (D7, D30 and D37) as compared to D0. Up- or downregulation were defined as values > 0 and < 0, respectively. The model included a random intercept for each subject and was blocked by gender. The t-statistics were used to calculate p-values and Benjamini-Hochberg FDR-adjusted *p-*values. Intergroup comparisons made at D7, D30 and D37 were embedded in the model. Genes were deemed differentially expressed if FDR < 0.05. Determination of enrichment of genes belonging to the BTMs [39] (FDR *q* < 0.05) in the DEG for each contrast was performed by hypergeometric test. The identification of the BTM was confirmed on condition that the RNA expression of the majority of genes within the BTM were significantly different from baseline (using the FDR *p-*values). Up- or downregulation of a BTM was determined by the relative prevalence of genes with RNA expression significantly higher or lower than baseline, respectively. BTMs were grouped based on prior knowledge and on the analysis of genes underlying their enrichment. The overall gene expression for any particular group of BTM was summarized by applying PCA to a matrix describing the gene expression of all genes underlying the BTM expression (irrespective of whether they were differentially expressed at the group level) for each subject, and then using the scores in PC1.

Multilevel PCA was applied in order to take advantage of the repeated measurement structure of the data, and to highlight the treatment effect within the subjects separately from the biological variation between subjects. To this purpose, we first decomposed the within-subject variation in the dataset, then applied PCA on the within-subject variation matrix. Multilevel PCA analysis was performed using the *mixOmics* package in R.

### Statistical analyses

To assess the relationship between antibody responses (y) and factors such as timepoint post vaccination, or gene expression, we used linear mixed models with random intercept for each subject (package lme4 v. 1.1–21 [60]). The Akaike information criterion was used for model selection. In all cases tested, including an interaction term did not increase the model performance, hence no interaction term was included. *P*-values for fixed effects were obtained using the ANOVA function from the R package lmerTest v.3.1–0 [61]. For the analysis of the relationship between antibody responses and gene expression, we did not include values below LOD. Assumption of linearity of the response, homoscedasticity and normality of the residuals were assessed visually, and met. Qualitative analysis refers to set analysis using Venn diagrams without direct hypothesis testing supported by statistical analysis.

Transcriptional response heterogeneity within a participant group by post-vaccination timepoint was visualized by reverse cumulative distribution analysis performed in R, as well as evaluated in terms of Jaccard similarity by a method adapted from ref. [62]. Briefly, using FC > 2 as cut-off for up/down-regulation, we calculated for all possible pairs of individuals within a single group, the proportion of genes that were commonly up- or down-regulated. The JSI value was calculated as ratio between the genes commonly up- or down-regulated, and the genes up- or down-regulated in either of the two considered subjects (i.e., JSI value = $\frac{A\cap B}{A\cup B}$).

## Supporting information

S1 Fig Genes shared between groups at 1 week post dose 2. (PDF)

S2 Fig B cell associated gene expression at D37 correlates with antibody levels. (PDF)

S3 Fig Similar trends in functional gene expression across the participant groups. (PDF)

S4 Fig Kinetics of the anti-M72 antibody response as a function of PC1 levels. (PDF)

S5 Fig Heatmap presentation of individual transcriptional responses. (PDF)

S1 Table List of genes in the intersections of the Venn Diagram in S1 Fig. (DOCX)

S2 Table Group level data as presented in Fig 2B. (DOCX)

S3 Table Genes underlying each module enrichment in Fig 5. (DOCX)
