# Supplementary material for: Subsequent AS01-adjuvanted vaccinations induce similar transcriptional responses in populations with different disease statuses
Source: PLoS One. 2022 Nov 10;17(11):e0276505. doi: 10.1371/journal.pone.0276505 (PMC9648731; doi:10.1371/journal.pone.0276505)
Supplement: S1 Fig — (PDF) [file pone.0276505.s001.pdf]

S1 Figure

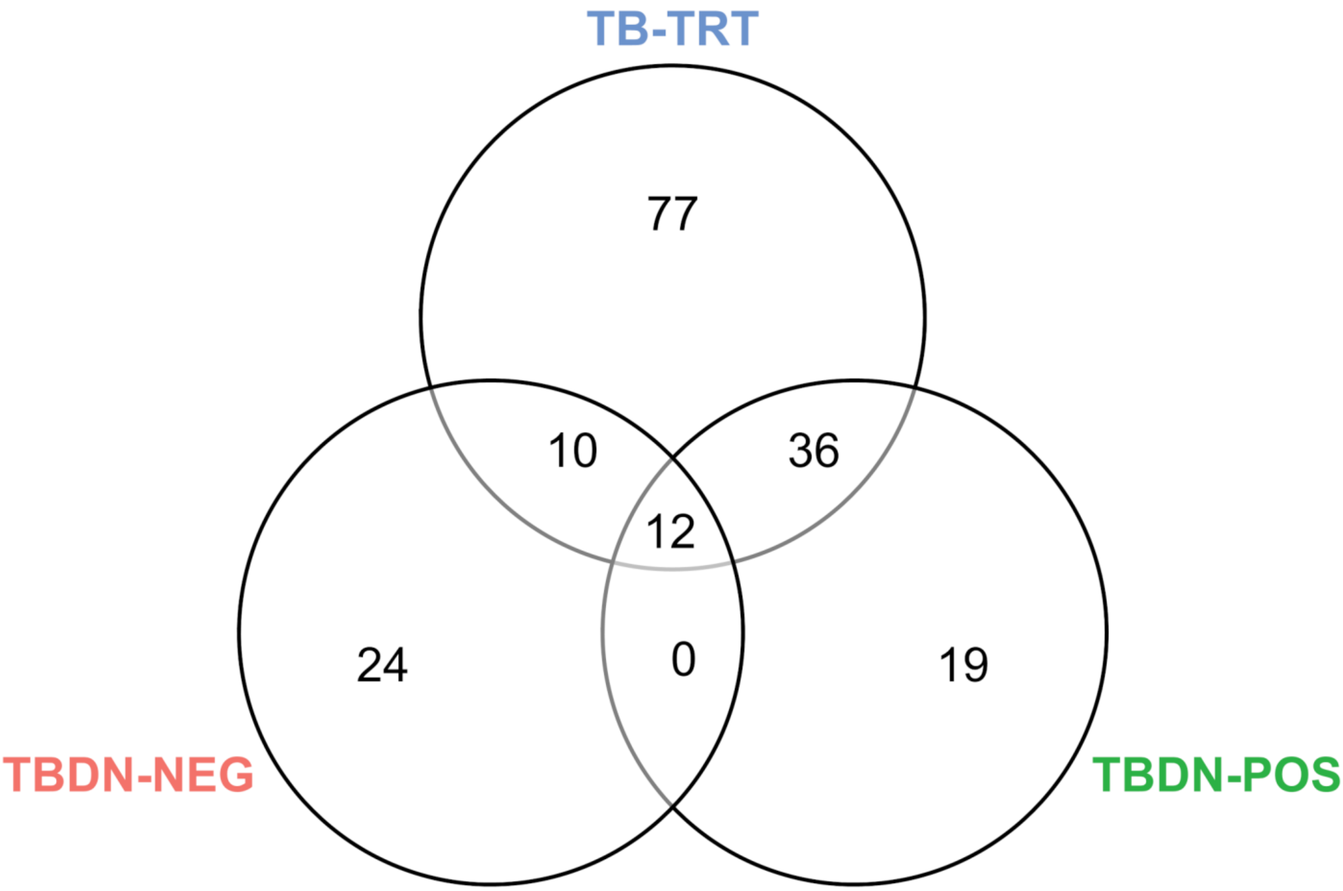

Genes shared between groups at 1 week post-dose 2. Numbers of differently expressed genes (DEG) shared between the participant groups at 1 week post-dose 2 are presented as Venn diagram. TBDN-POS/NEG, tuberculosis disease-naïve, purified protein derivative-positive/negative. TB-TRT, tuberculosis-treated.
