# Supplementary material for: Subsequent AS01-adjuvanted vaccinations induce similar transcriptional responses in populations with different disease statuses
Source: PLoS One. 2022 Nov 10;17(11):e0276505. doi: 10.1371/journal.pone.0276505 (PMC9648731; doi:10.1371/journal.pone.0276505)
Supplement: S2 Fig — (PDF) [file pone.0276505.s002.pdf]

S2 Figure

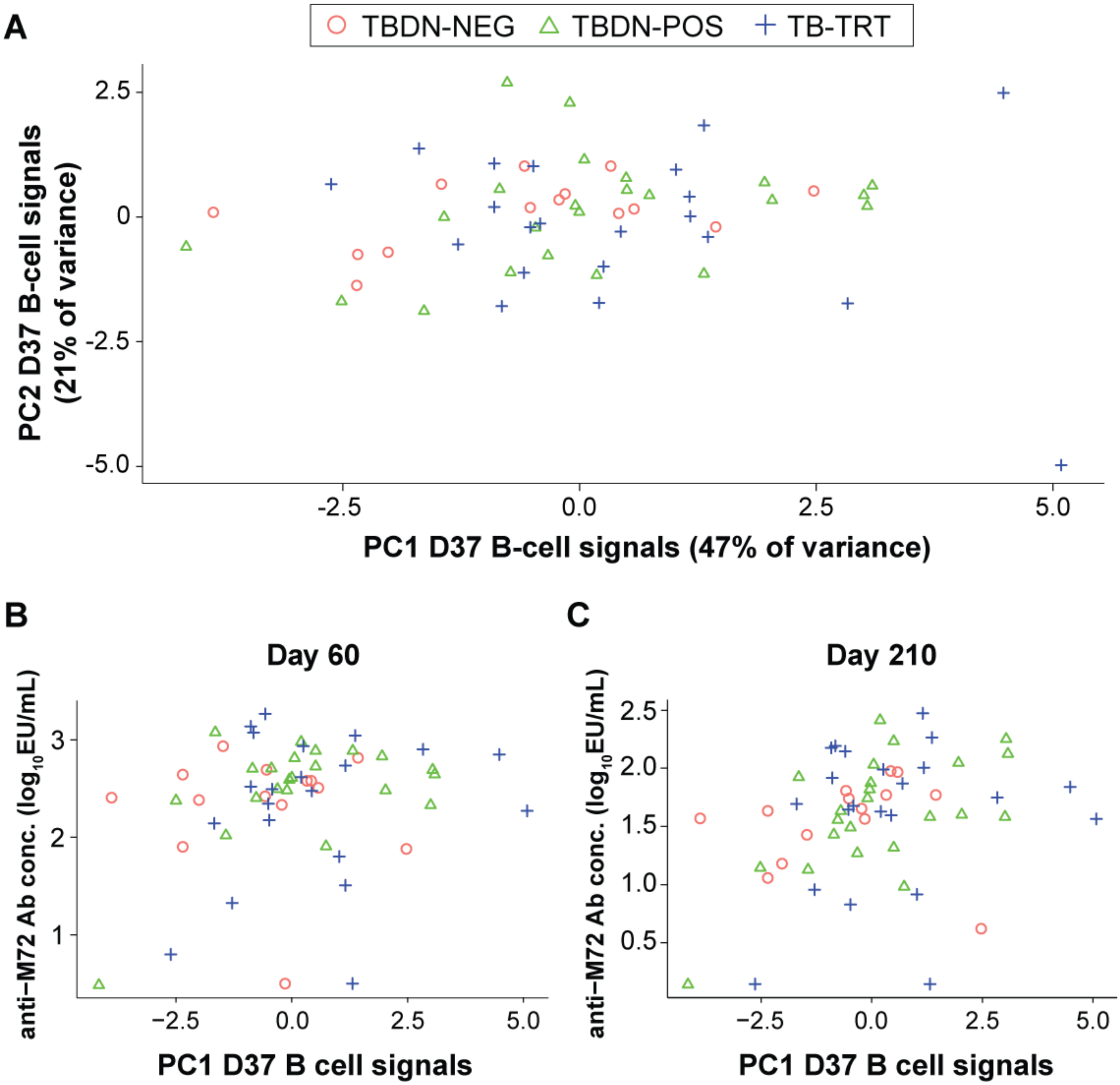

B-cell associated gene expression at D37 correlates with antibody levels. Principal component (PC) analysis was performed on expression values (in fold changes at Day [D]37 over D0) for genes assigned to the five B-cell associated blood transcription modules (no. 4 in Fig. 5). Each symbol in A-C represents one participant. (A) Scatter plots of the individual D37 PC1 and PC2 scores are presented. (B and C) The PC1 scores were plotted against the log10 anti-M72 antibody concentrations (expressed in Elisa Units [EU]/ml) measured at D60 (B) or D210 (C) are shown.
