# Supplementary material for: Subsequent AS01-adjuvanted vaccinations induce similar transcriptional responses in populations with different disease statuses
Source: PLoS One. 2022 Nov 10;17(11):e0276505. doi: 10.1371/journal.pone.0276505 (PMC9648731; doi:10.1371/journal.pone.0276505)
Supplement: S3 Fig — (PDF) [file pone.0276505.s003.pdf]

S3 Figure

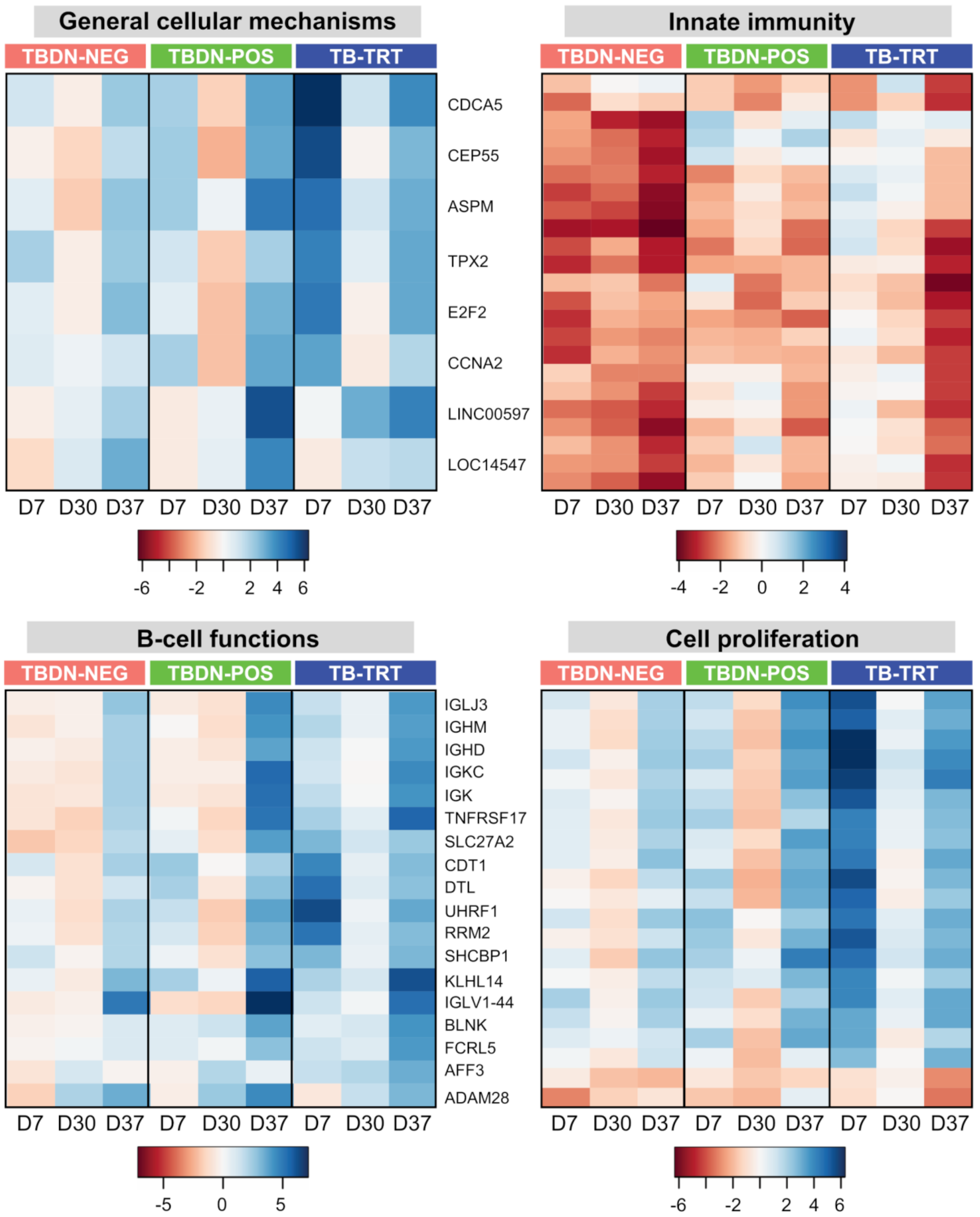

Similar trends in functional gene expression across the participant groups. Heatmaps representing RNA expression values of the genes (rows) underlying the enrichment of the four identified module categories (Fig. 4) are presented by participant group and timepoint (columns). Values are expressed in log2 average fold change over baseline (Day 0), with the level of upregulation (blue) or downregulation (red) color-coded according to the key below each heatmap. Gene names are described to the right of the heatmaps. TBDN-POS/NEG, tuberculosis disease-naïve, purified protein derivative-positive/negative participant groups. TB-TRT, tuberculosis-treated participant group.
