# Supplementary material for: Subsequent AS01-adjuvanted vaccinations induce similar transcriptional responses in populations with different disease statuses
Source: PLoS One. 2022 Nov 10;17(11):e0276505. doi: 10.1371/journal.pone.0276505 (PMC9648731; doi:10.1371/journal.pone.0276505)
Supplement: S4 Fig — (PDF) [file pone.0276505.s004.pdf]

S4 Figure

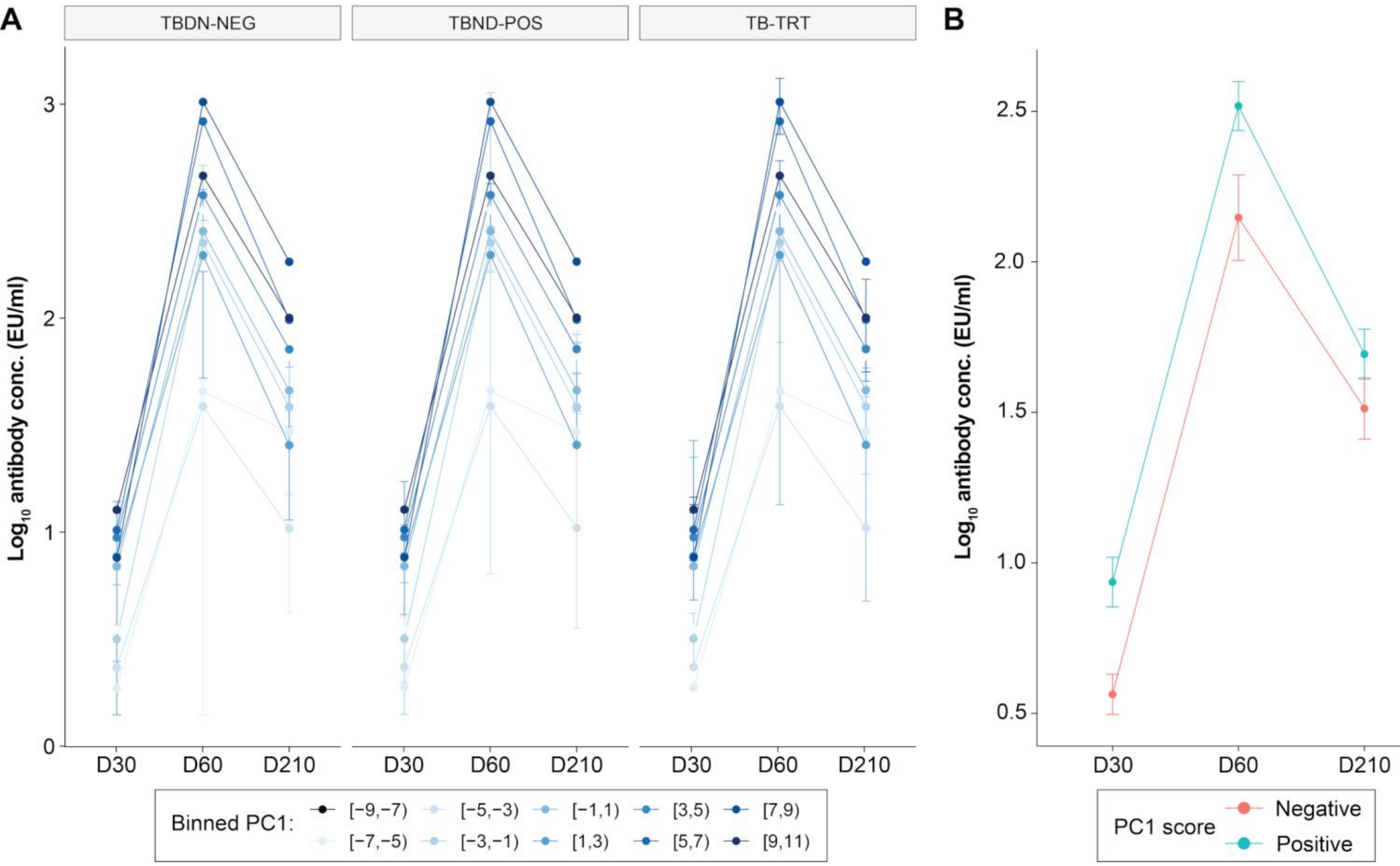

Kinetics of anti-M72 antibody responses as a function of PC1 levels. Responses (expressed as log10 concentrations in Elisa Units (EU)/mL) at Day (D)30, D60 and D210 are presented for the tuberculosis disease-naïve (TBDN) purified protein derivative (PPD)-negative (TBDN-NEG), TBDN PPD-positive (TBDN-POS), and tuberculosis-treated (TB-TRT) groups. First principal component (PC1) scores representing the response levels were binned (i.e. integrated over discrete intervals) and color-coded, as indicated in the key below the plots. Each colored line represents an individual subject (A, presented by group) or all subjects across groups with the PC1 score indicated (B).
