# Supplementary material for: Subsequent AS01-adjuvanted vaccinations induce similar transcriptional responses in populations with different disease statuses
Source: PLoS One. 2022 Nov 10;17(11):e0276505. doi: 10.1371/journal.pone.0276505 (PMC9648731; doi:10.1371/journal.pone.0276505)
Supplement: S5 Fig — (PDF) [file pone.0276505.s005.pdf]

S5 Figure

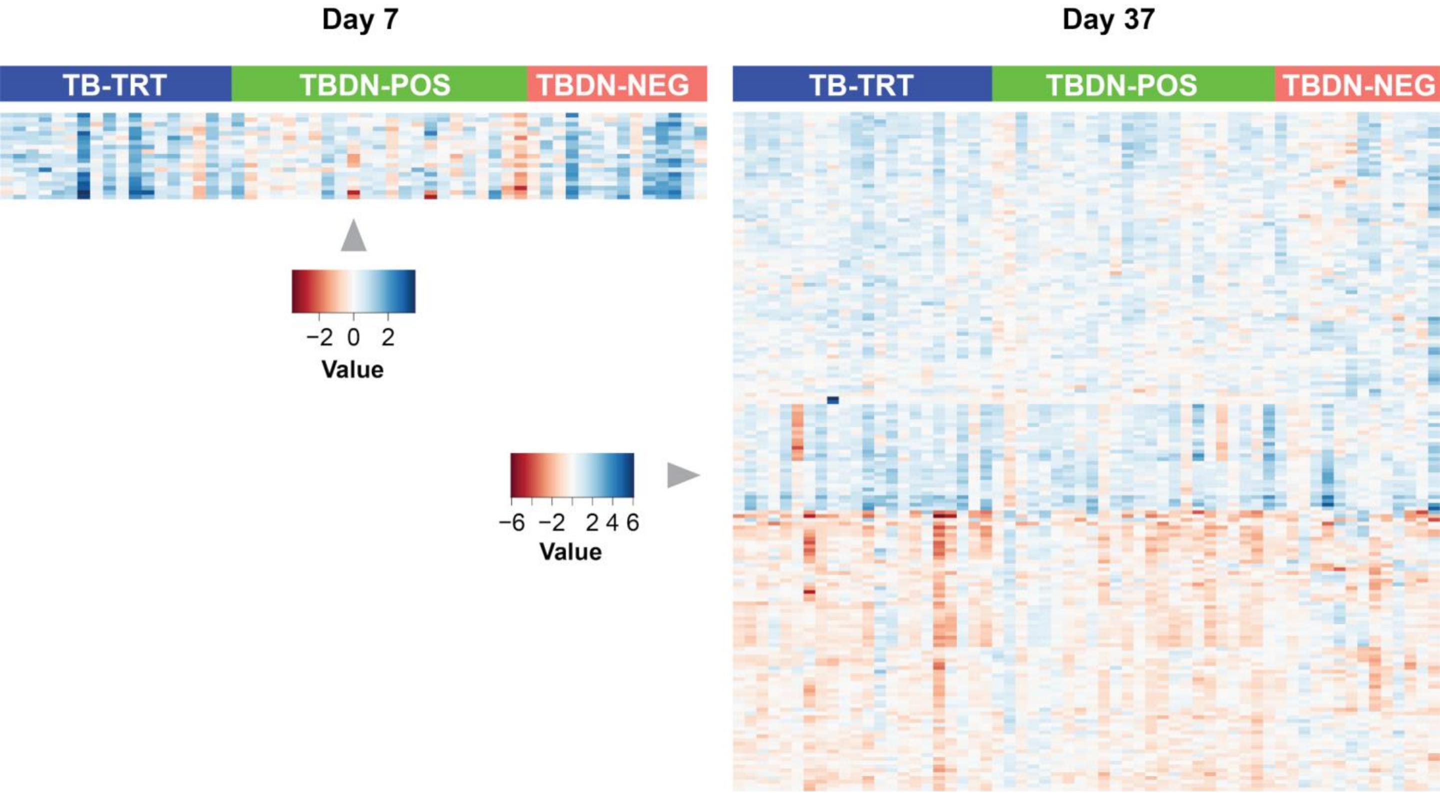

Heatmap presentation of individual transcriptional responses. Individual log2 fold changes for individual DEG (rows) at Day (D)7 (left) and D37 (right) vs D0 are presented. Values are color-coded according to the keys below each heatmap
