# Supplementary material for: Subsequent AS01-adjuvanted vaccinations induce similar transcriptional responses in populations with different disease statuses
Source: PLoS One. 2022 Nov 10;17(11):e0276505. doi: 10.1371/journal.pone.0276505 (PMC9648731; doi:10.1371/journal.pone.0276505)
Supplement: S1 Table — (DOCX) [file pone.0276505.s006.docx]

**S1 Table. List of genes in the intersections of the Venn Diagram in S1 Figure**

| **TBDN-NEG** | **TBDN-POS** | **TBT** | **TBDN-NEG** ∩ **TBT** | **TBDN-POS ∩ TBT** | **TBDN-POS ∩ TBT ∩ TBDN-NEG** |
| --- | --- | --- | --- | --- | --- |
| ABCA6 | **CAV1** | ABCA1 | CFB | ANK3 | **ADAM28** |
| BISPR | CCNA2 | ABTB2 | CXCL10 | ANKRD36BP2 | AOPEP |
| CCL23 | CELF2 | ADAMTSL4 | EDN1 | ASPM | DLG1 |
| CLGN | CEP55 | **AFF3** | FJX1 | ATP8A1 | **IGLC1** |
| CMPK2 | **COBLL1** | AKT3 | IFIT3 | BIRC5 | **IGLV1-44** |
| HCG4 | HS3ST3B1 | APOL6 | LINC01465 | **BLNK** | LINC01366 |
| IFIT5 | LOC145474 | AVL9 | NEURL3 | BMS1P20 | **MZB1** |
| IFNB1 | LRRC8C | **BACH2** | SPRED1 | CDCA5 | **PSMG4** |
| IL36G | MKI67 | **BBX** | TNF | **DTNB-AS1** | **SLC6A16** |
| LAMP3 | NSUN7 | **BHLHE41** | **TRMT2B** | **FBXO22** | **TTLL5** |
| OAS1 | POLR1F | C2CD4B |  | FBXO32 | **ZCCHC7** |
| OAS2 | S100Z | C5AR2 |  | GLDC | ZHX2 |
| OAS3 | SLC27A2 | CCR2 |  | **IGHD** |  |
| PRRG4 | **SLC2A5** | CD274 |  | **IGHM** |  |
| RSAD2 | SV2C | **CD38** |  | **IGK** |  |
| RTP4 | SYTL3 | CDT1 |  | **IGKC** |  |
| SAMD9L | TCF12 | CHPT1 |  | **IGLJ3** |  |
| SGPP2 | UBE2QL1 | CLDN23 |  | **IGLV@** |  |
| STAT1 | ZNF215 | CLIC2 |  | KIF18B |  |
| SYNPO2 |  | **COCH** |  | **KLHL14** |  |
| TFPI2 |  | **COL4A4** |  | **LARGE-AS1** |  |
| TRIM5 |  | DLGAP5 |  | LINC00597 |  |
| TSLP |  | DOCK9 |  | LOC101929774 |  |
| XAF1 |  | E2F2 |  | LRRC2 |  |
|  |  | EIF2AK4 |  | MIA2 |  |
|  |  | EIF4E |  | MPV17L |  |
|  |  | EMP1 |  | PCLAF |  |
|  |  | ESR1 |  | **PIP5K1B** |  |
|  |  | ETS2 |  | PTPN9 |  |
|  |  | FAM30A |  | **RPS27** |  |
|  |  | **FCRL5** |  | RRM2 |  |
|  |  | **GEN1** |  | SEPTIN7 |  |
|  |  | ICAM1 |  | SH3GL3 |  |
|  |  | IFI6 |  | **SLC38A9** |  |
|  |  | IFIT2 |  | **TNFRSF17** |  |
|  |  | **IL6** |  | UHRF1 |  |
|  |  | **KCNH8** |  |  |  |
|  |  | KCNJ2-AS1 |  |  |  |
|  |  | **KCNQ5** |  |  |  |
|  |  | KDM3A |  |  |  |
|  |  | **KMO** |  |  |  |
|  |  | LAG3 |  |  |  |
|  |  | LGALS3 |  |  |  |
|  |  | **LMBRD1** |  |  |  |
|  |  | LOC100506098 |  |  |  |
|  |  | LOC101927811 |  |  |  |
|  |  | LOC644090 |  |  |  |
|  |  | **LUC7L3** |  |  |  |
|  |  | MCTP2 |  |  |  |
|  |  | MS4A4A |  |  |  |
|  |  | MUC6 |  |  |  |
|  |  | MXD1 |  |  |  |
|  |  | NLRC4 |  |  |  |
|  |  | OASL |  |  |  |
|  |  | PPARGC1A |  |  |  |
|  |  | PROM1 |  |  |  |
|  |  | RIN2 |  |  |  |
|  |  | RNF144B |  |  |  |
|  |  | SDHD |  |  |  |
|  |  | SEPTIN7P14 |  |  |  |
|  |  | SF1 |  |  |  |
|  |  | **SHCBP1** |  |  |  |
|  |  | SOCS3 |  |  |  |
|  |  | SRSF1 |  |  |  |
|  |  | SRSF4 |  |  |  |
|  |  | **SSPN** |  |  |  |
|  |  | **STRBP** |  |  |  |
|  |  | TACSTD2 |  |  |  |
|  |  | **TEX9** |  |  |  |
|  |  | TP63 |  |  |  |
|  |  | TPX2 |  |  |  |
|  |  | TSHZ2 |  |  |  |
|  |  | UICLM |  |  |  |
|  |  | UPB1 |  |  |  |
|  |  | USP9Y |  |  |  |
|  |  | VMO1 |  |  |  |
|  |  | ZFY |  |  |  |

Only intersections with data are shown. Gene symbols in bold are expressed in at least 2 datasets from the Human Protein Atlas (Uhlén M et al., Tissue-based map of the human proteome. Science. 2015; 347:1260419. doi: 10.1126/science.1260419). TBDN-POS/NEG, tuberculosis disease-naïve, purified protein derivative-positive/negative participant groups. TB-TRT, tuberculosis-treated participant group.
