# Supplementary material for: Subsequent AS01-adjuvanted vaccinations induce similar transcriptional responses in populations with different disease statuses
Source: PLoS One. 2022 Nov 10;17(11):e0276505. doi: 10.1371/journal.pone.0276505 (PMC9648731; doi:10.1371/journal.pone.0276505)
Supplement: S2 Table — (DOCX) [file pone.0276505.s007.docx]

**S2 Table. Group level data as presented in Figure 2B**

| **Timing** | **Group** | **N** | **Log_10_ anti-M72 IgG2 concentration (EU/mL)** | | | |
| --- | --- | --- | --- | --- | --- | --- |
|  |  |  | **Values** | **SD** | **SE** | **CI** |
| PRE | TBDN-NEG | 15 | 0.5233 | 0.2863 | 0.0739 | 0.1586 |
|  | TBDN-POS | 25 | 0.7098 | 0.3319 | 0.0664 | 0.1370 |
|  | TB-TRT | 24 | 1.0543 | 0.4728 | 0.0965 | 0.1996 |
| PI (D30) | TBDN-NEG | 15 | 1.5689 | 0.2719 | 0.0702 | 0.1506 |
|  | TBDN-POS | 25 | 1.6434 | 0.2265 | 0.0453 | 0.0935 |
|  | TB-TRT | 22 | 1.6075 | 0.3064 | 0.0653 | 0.1358 |
| PII (D60) | TBDN-NEG | 15 | 2.2289 | 0.4754 | 0.1228 | 0.2633 |
|  | TBDN-POS | 25 | 2.4252 | 0.3469 | 0.0694 | 0.1432 |
|  | TB-TRT | 22 | 2.2318 | 0.4930 | 0.1051 | 0.2186 |
| PII (D210) | TBDN-NEG | 15 | 0.1691 | 0.2795 | 0.0722 | 0.1548 |
|  | TBDN-POS | 24 | 0.1461 | 0.3380 | 0.0690 | 0.1427 |
|  | TB-TRT | 24 | 0.1461 | 0.4563 | 0.0931 | 0.1927 |

Within-subject standard deviation (SD), standard error (SE) and confidence intervals (CI) are shown. Data were processed to take into account the presence of within-subject variables, as described in Morey, R. D. (2008), Confidence intervals from normalized data: a correction to Cousineau (2005), Tutorials in Quantitative Methods for Psychology, 4, 61–64. doi: 10.20982/tqmp.04.2.p061. TBDN-POS/NEG, tuberculosis disease-naïve, purified protein derivative-positive/negative participant groups. TB-TRT, tuberculosis-treated participant group.
