# Supplementary material for: Subsequent AS01-adjuvanted vaccinations induce similar transcriptional responses in populations with different disease statuses
Source: PLoS One. 2022 Nov 10;17(11):e0276505. doi: 10.1371/journal.pone.0276505 (PMC9648731; doi:10.1371/journal.pone.0276505)
Supplement: S3 Table — (DOCX) [file pone.0276505.s008.docx]

**S3 Table. Genes underlying each module enrichment in Fig. 5**

| **BTM ID** | **Genes underlying enrichment** | | | | | | | | | | | | | | |  |
| --- | --- | --- | --- | --- | --- | --- | --- | --- | --- | --- | --- | --- | --- | --- | --- | --- |
| LI.M246 | LINC00597 | LOC145474 |  |  |  |  |  |  |  |  |  |  |  |  |  |  |
| LI.M4.0 | TPX2 | DTL | KIF18B | PBK | MKI67 | ASPM | CDT1 | UHRF1 | DHFR | BIRC5 | CEP55 | RRM2 | CC2 | CDCA5 | NEIL3 |  |
| LI.M4.1 | TPX2 | DTL | PBK | CDT1 | ASPM | UHRF1 | MKI67 | DHFR | BIRC5 | CEP55 | RRM2 | CC2 | CDCA5 | KIF18B | CCNB2 |  |
| LI.M4.4 | CDT1 | TPX2 |  |  |  |  |  |  |  |  |  |  |  |  |  |  |
| LI.M4.5 | CEP55 | CDCA5 | RRM2 | UHRF1 |  |  |  |  |  |  |  |  |  |  |  |  |
| LI.M4.7 | CCNB2 | CC2 | CDT1 |  |  |  |  |  |  |  |  |  |  |  |  |  |
| LI.M4.10 | CCNB2 | DTL | RRM2 |  |  |  |  |  |  |  |  |  |  |  |  |  |
| LI.M46 | DTL | RRM2 | CDT1 | UHRF1 | SLC27A2 |  |  |  |  |  |  |  |  |  |  |  |
| LI.M103 | CC2 | CCNB2 | E2F2 |  |  |  |  |  |  |  |  |  |  |  |  |  |
| LI.M13 | CXCL10 |  |  |  |  |  |  |  |  |  |  |  |  |  |  |  |
| LI.M29 | TNF |  |  |  |  |  |  |  |  |  |  |  |  |  |  |  |
| LI.M38 | TNF |  |  |  |  |  |  |  |  |  |  |  |  |  |  |  |
| LI.M47.0 | IGHM | KLHL14 | IGHD | ADAM28 | IGLJ3 |  |  |  |  |  |  |  |  |  |  |  |
| LI.M47.1 | IGK | BLNK | KLHL14 | IGHM |  |  |  |  |  |  |  |  |  |  |  |  |
| LI.M47.2 | IGK | IGKC | IGHD | IGLV1-44 |  |  |  |  |  |  |  |  |  |  |  |  |
| LI.M68 | TNF | CXCL10 |  |  |  |  |  |  |  |  |  |  |  |  |  |  |
| LI.M75 | RSAD2 | OAS1 | OAS3 | CXCL10 |  |  |  |  |  |  |  |  |  |  |  |  |
| LI.M78 | EDN1 |  |  |  |  |  |  |  |  |  |  |  |  |  |  |  |
| LI.M111.1 | CXCL10 |  |  |  |  |  |  |  |  |  |  |  |  |  |  |  |
| LI.M127 | STAT1 | RSAD2 |  |  |  |  |  |  |  |  |  |  |  |  |  |  |
| LI.M150 | OAS1 | OAS3 | RSAD2 |  |  |  |  |  |  |  |  |  |  |  |  |  |
| LI.M156.0 | IGKC | ADAM28 | IGHD | TNFRSF17 | IGLV1-44 | IGLJ3 |  |  |  |  |  |  |  |  |  |  |
| LI.M156.1 | TNFRSF17 | IGK | IGKC | IGHD | IGHM | IGLV1-44 | IGLJ3 |  |  |  |  |  |  |  |  |  |
| LI.M165 | IFIT3 | RSAD2 | PRRG4 | LAMP3 |  |  |  |  |  |  |  |  |  |  |  |  |
| LI.S5 | PRRG4 | ABCA6 |  |  |  |  |  |  |  |  |  |  |  |  |  |  |
| LI.S10 | ABCA6 |  |  |  |  |  |  |  |  |  |  |  |  |  |  |  |
| LI.S11 | SGPP2 | PRRG4 |  |  |  |  |  |  |  |  |  |  |  |  |  |  |
| LI.M4.2 | CEP55 | TPX2 | ASPM |  |  |  |  |  |  |  |  |  |  |  |  |  |
| LI.M4.14 | E2F2 | CEP55 |  |  |  |  |  |  |  |  |  |  |  |  |  |  |

BTM, blood transcriptional module as described in Li et al. Molecular signatures of antibody responses derived from a systems biology study of five human vaccines. Nat Immunol. 2014; 15: 195-204
